# Profiling Tryptophan Catabolites of Human Gut Microbiota and Acute-Phase Protein Levels in Neonatal Dried Blood Specimens

**DOI:** 10.3389/fmicb.2021.665743

**Published:** 2021-10-27

**Authors:** Anne-Christine Aust, Eliska Benesova, Veronika Vidova, Katerina Coufalikova, Sona Smetanova, Ivo Borek, Petr Janku, Eva Budinska, Jana Klanova, Vojtech Thon, Zdenek Spacil

**Affiliations:** ^1^RECETOX, Faculty of Science, Masaryk University, Brno, Czechia; ^2^Department of Pediatrics, University Hospital Brno and Masaryk University Medical School, Brno, Czechia; ^3^Department of Gynecology and Obstetrics, University Hospital Brno and Masaryk University Medical School, Brno, Czechia

**Keywords:** human gut microbiota, tryptophan and kynurenine metabolism, dried blood specimens, acute-phase proteins, immunomodulation

## Abstract

National screening programs use dried blood specimens to detect metabolic disorders or aberrant protein functions that are not clinically evident in the neonatal period. Similarly, gut microbiota metabolites and immunological acute-phase proteins may reveal latent immune aberrations. Microbial metabolites interact with xenobiotic receptors (i.e., aryl hydrocarbon and pregnane-X) to maintain gastrointestinal tissue health, supported by acute-phase proteins, functioning as sensors of microbial immunomodulation and homeostasis. The delivery (vaginal or cesarean section) shapes the microbial colonization, which substantially modulates both the immune system’s response and mucosal homeostasis. This study profiled microbial metabolites of the kynurenine and tryptophan pathway and acute-phase proteins in 134 neonatal dried blood specimens. We newly established neonatal blood levels of microbial xenobiotic receptors ligands (i.e., indole-3-aldehyde, indole-3-butyric acid, and indole-3-acetamide) on the second day of life. Furthermore, we observed diverse microbial metabolic profiles in neonates born vaginally and *via* cesarean section, potentially due to microbial immunomodulatory influence. In summary, these findings suggest the supportive role of human gut microbiota in developing and maintaining immune system homeostasis.

## Introduction

Dried blood specimens (DBS) are used to quantify circulating levels of drugs (Kloosterboer et al., 2018), metabolites (Sain-van der Velden et al., 2017), and proteins. The advantages over a conventional blood draw include minimally invasive sampling, suitable for neonates and other vulnerable populations, fewer processing and handling steps, and facile storage. Neonatal DBS collected from a heel prick are widely used in nationwide neonatal screening programs for inherited endocrine and metabolic disorders (Mechtler et al., 2012).

The initial exposure to microbiota during and immediately after birth influences the lifelong colonization and modulates the innate and adaptive immune system (Neu and Rushing, 2011), potentially causing a decreased tolerance or an exorbitant antigen representation, inflammatory response, and damage to the mucosal barrier function (Francino, 2018). Natural vaginal delivery (VD) or cesarean delivery (CD) shapes the diversity of commensal, symbiotic, and pathogenic microorganisms colonizing the human body, collectively referred to as the microbiota (Penders et al., 2006; Palmer et al., 2007; Mitsou et al., 2008; Wall et al., 2009). The composition and timing of gut microbiota colonization vary in VD and CD neonates (Bennet and Nord, 1987; Grönlund et al., 1999; Penders et al., 2006; Palmer et al., 2007; Mitsou et al., 2008). Fecal and vaginal microbiota dominate the initial colonization in VD neonates (Mändar and Mikelsaar, 1996; Bezirtzoglou, 1997; Grönlund et al., 1999; Matsumiya et al., 2002; Wall et al., 2009). For instance, microaerophilic *Lactobacillus* species (*ca.* 25% of total microbiota) frequently colonizes VD infants (Matsumiya et al., 2002; Wall et al., 2009). On the other hand, CD neonates are primarily exposed to nosocomial bacteria or topical skin microbiota (Bezirtzoglou, 1997; Grönlund et al., 1999; Mackie et al., 1999; Wall et al., 2009). CD infants’ gut microbiota typically contain a smaller share of strict anaerobes such as *Bacteriodetes fragilis* and *Bifidobacteria* (Bennet and Nord, 1987; Grönlund et al., 1999; Torun et al., 2002; Penders et al., 2006; Wall et al., 2009). The initial microbial composition’s nuances can modulate the immune system’s development and affect the infant’s subsequent health (Francino, 2018; Wampach et al., 2018).

**Figure 1:**
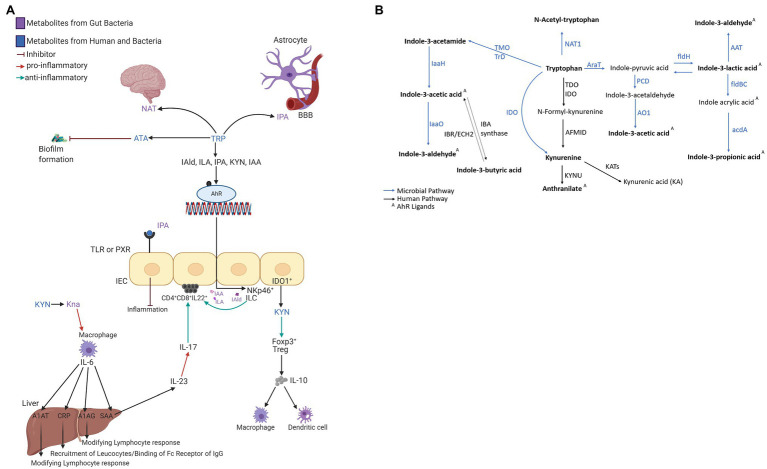
**(A)** The interaction network of microbial tryptophan catabolites and acute-phase proteins mediating the anti-inflammatory and immune-supportive function. **(B)** Microbial and human co-metabolism. Created with BioRender.com.

The circulating microbial metabolites reflect the diversity of human gut microbiota and endogenous inflammatory markers (i.e., acute-phase proteins—APP) the immune system’s reaction. The microbial community builds contact with intestinal epithelial immune cells’ receptors and stimulates signaling cascades leading to cell differentiation and inflammatory response control (Romagnani, 2006). Microbial catabolites of aromatic amino acids (e.g., tryptophan) act protectively in the host immune homeostasis, similar to short-chain fatty acids (i.e., acetate, butyrate, and propionate; Jin et al., 2017; Pavlova et al., 2017). Tryptophan catabolites, i.e., indole-3-acetic acid (IAA; Elsden et al., 1976; Smith and Macfarlane, 1996; Finegold et al., 2002; Russell et al., 2013; Roager and Licht, 2018), indole-3-lactic acid (ILA; Aragozzini et al., 1979; Smith and Macfarlane, 1996; Russell et al., 2013; Honoré et al., 2016; Cervantes-Barragan et al., 2017; Dodd et al., 2017; Roager and Licht, 2018), and indole-3-propionic acid (IPA; Elsden et al., 1976; Wikoff et al., 2009; Williams et al., 2014; Dodd et al., 2017; Wlodarska et al., 2017; Roager and Licht, 2018), interact with xenobiotic receptors (i.e., aryl hydrocarbon receptor, AHR, and pregnane X receptor, PXR; Roager and Licht, 2018). The AHR is a ligand-activated transcriptional factor widely expressed in immune cells that attenuates autoimmune responses and ensures gastrointestinal tissue health (Figure 1A). The ligand-specific activation of the AHR signaling pathway is immunomodulatory to the host. Microbial tryptophan catabolites regulate the production of pro-inflammatory cytokines (i.e., INF-γ and IL-2) in TH1 cells and anti-inflammatory cytokines (i.e., IL-10) in TH2 cells (Mangge et al., 2014; Alexeev et al., 2016; Lanis et al., 2017; Gao et al., 2018; Krishnan et al., 2018). Microbial AHR ligands modulate intestinal barrier function and the resistance against enteric pathogens (Liu et al., 2018). Indole-3-acetamide (IAM) and indole-3-butyric acid (IBA) are precursors of IAA (Figure 1B; Agus et al., 2018). Indole-3-aldehyde (IAld), IAA, IAM, and ILA activate ILC3 through AHR signaling, producing IL-22 to induce resistance against mucosal candidiasis (Zelante et al., 2013; Zhang et al., 2019). IBA, the metabolic product of Clostridia species (Lombard and Dowell, 1983), occurs in human urine (Tonelli et al., 1982) and plasma. IBA cooccurred with the incidence of inflammatory bowel syndrome (IBS) in schizophrenic patients (Cai et al., 2012). A potential mechanism to control inflammation by IBA and IAA is competitive inhibition of phospholipase A_2_ (Dileep et al., 2013). ILA reprograms intraepithelial CD4^+^T cells in immunoregulatory CD4^+^CD8_αα_^+^ (Cervantes-Barragan et al., 2017). *In vitro* studies in gram-positive and gram-negative bacterial cell cultures (*Bacillus subtilis*, *Pseudomonas aeruginosa*, *Salmonella enterica*, and *Staphylococcus aureus*) demonstrated biofilm formation inhibition by anthranilate (ATA; Li et al., 2017). The interaction between microbial metabolites and the neonates’ immune system emphasizes their role in the epithelial barrier function and reveals their importance in the signaling cascade of the immune systems’ local and systemic response.

The quantitative profiling of circulating acute-phase proteins (APPs) monitors the systemic inflammatory response (Vidova et al., 2019). APPs are not transportable across the placental barrier and represent a surrogate for the neonate’s innate immune system’s activation. C-reactive protein (CRP) and serum amyloid A (SAA) are synthesized in hepatocytes (Figure 1A) after stimulation by cytokines (i.e., IL-6, IL-1, IL-8, and TNF-α; Heinrich et al., 1990). In healthy individuals, circulating SAA1, SAA2, and CRP levels are low but increase between 10-fold and 1,000-fold during the acute phase of inflammation (Clyne and Olshaker, 1999; Haran et al., 2013; Buck et al., 2016). SAA1 is arguably a more reliable inflammation marker than CRP as SAA levels rise earlier, more rapidly, and have higher amplitude (Arnon et al., 2007). SAA4 is a constitutive apolipoprotein with a stable blood concentration during the acute phase of inflammation (Yamada et al., 1997; Buck et al., 2016). The initial microbial colonization influenced by the mode of delivery induces measurable perturbations in APPs. Higher concentrations of SAA1 and CRP were reported in VD compared to CD neonates (Marchini et al., 2000). Blood levels of A1AT, A1AG1, and A1AG2 increase by several folds in response to inflammation (Hochepied et al., 2003; Janciauskiene et al., 2011).

In this study, we profiled nine tryptophan catabolites (i.e., ATA, IAA, IAM, IAld, IBA, ILA, IPA, kynurenine—KYN, and N-acetyl tryptophan—NAT) and seven APPs (i.e., A1AT, A1AG1, A1AG2, SAA1, SAA1/2, SAA4, and CRP) in a DBS punch (3mm or 1/8″, equivalent to 3μl of blood). We analyzed 134 neonatal DBSs from a birth cohort study: Central European Longitudinal Studies of Parents and Children – The Next Generation (CELSPAC-TNG) to explore early-life immunomodulation attributed to the metabolism of the human gut microbiota. We explored the delivery mode’s influence on tryptophan catabolite and acute-phase protein profile.

## Materials and Methods

### Study Design

DBS samples from 134 neonates (20 delivered *via* cesarean section and 114 delivered vaginally) collected under IRB approval were part of the CELSPAC-TNG study at Faculty Hospital Brno (Ethical Committee CELSPAC/EK/4/2016, in 2016–2017). Characteristics of individual neonates, including gestational age, delivery mode, sex, birth weight, birth length, Apgar score, DBS sampling, and anamnesis, are shown in Supplementary Table S-1. The study subjects were female (*n*=56) and male (*n*=78), with an average birth weight of 3,494g and an average birth length of 50.5cm (Supplementary Tables S-1 and S-2). We show the average, minimal, and maximal values for birth length, weight, gestation age, Apgar score, and the delay from the birth to DBS sampling for VD and CD neonates separately in Supplementary Table S-2. For DBS sampling, a small amount of capillary blood from the heel prick was soaked into Whatman 903 filter paper and allowed to dry at room temperature for 3h. DBS punches (1/8″ or 3mm) were stored in the freezer at −80°C until analysis.

### Chemicals and Reagents

Isotopically labeled peptides used as internal standards for protein quantification were from JPT Technologies (Berlin, Germany). Sequences are listed in Supplementary Table S-3. The protein assay protocol was adapted from the previous study (Vidova et al., 2019). Isotopically labelled [^13^C_6_] indole-3-acetic acid (cat. #0317333), purity >97%, was from OlChemIm s.r.o. (Olomouc, Czech Republic). Isotopically labeled [^13^C_11_] [^15^N_2_] L-tryptophan (cat. #574597), purity ≥98%, was from Sigma-Aldrich (St. Louis, United States). Isotopically labeled [^2^D_4_] L-kynurenine (cat. #DLM-7842-PK), purity of 95%, was from Cambridge Isotope Laboratories, Inc. (Tewksbury, Massachusetts, United States). Isotopically labeled [^13^C_6_] anthranilic acid (cat. #PR-24225), purity 99%, was from Sigma-Aldrich (St. Louis, Massachusetts, USA). The chemical standard of _L_-tryptophan (cat. #51145; TraceCERT®), N-acetyl-tryptophan (cat. #PHR1177), indole-3-acetate (cat. #45533), purity 98%, serotonin (cat. #14927), purity≥98%, anthranilic acid (cat. #10680), purity≥99.5%, L-kynurenine (cat. #K8625), purity≥98%, were from Sigma-Aldrich (St. Louis, Massachusetts, USA). The chemical standard indole-3-carboxaldehyde (cat. #A15330), purity 99%, was from Alfa Aesar (Haverhill, Massachusetts, United States). The chemical standard indole-lactic-3- acid (≥97%; cat. #SC-255130), purity≥97%, was from Santa Cruz Biotechnology (Dallas, Texas, USA). Indole-3-butyric acid standard (≥99.0%; cat. #57310) was from Sigma-Aldrich (St. Louis, Massachusetts, United States). Liquid chromatography–mass spectrometry (LC–MS) grade acetonitrile (cat. #0013687802BS) and isopropanol (cat. #0016267802BS) were from Biosolv (Valkenswaard, Netherlands). Formic acid for mass spectrometry (cat. #94318) and ammonium bicarbonate BioUltra ≥99.5% (cat. #09830) were from Sigma-Aldrich (St. Louis, MO). BCA protein assay kit (cat. #23227) was from Thermo Fisher Scientist (Waltham, MA). Deionized water was produced using Millipore Simplicity 185 ultrapure water system (Merck Millipore corp., Billerica, MA).

### Metabolite Extraction

The complete DBS sample processing flowchart is shown in Supplementary Figure S-3. Based on material availability, one or two 3-mm DBS punches (equivalent to 3μl and 6μl of whole blood, respectively) were reconstituted in 150μl of 50mM ammonium bicarbonate buffer in an orbital shaker (1,600rpm, 60min). We removed a volume of 5μl for BCA (section Protein Extraction and Processing Protocol, and Mass Spectrometry Assays) and dried the remaining sample in a vacuum concentrator centrifuge (Savant SPD121 P SpeedVac, Thermo Fisher). A volume of 400μl of 80% isopropanol (*v/v*) was added to a dry sample and vortexed at 200rpm for 20min. The sample was briefly centrifuged, supernatant quantitatively transferred into a 96-well plate, and dried in the SpeedVac. Dry extracts were redissolved in 10μl 5% isopropanol (*v/v*) containing isotopically labeled standards: 200nM [^13^C_6_] indole-3-acetate, 2000nM [^2^H_5_] L-kynurenine, 20,000nM [^13^C_11_][^15^N_2_] L-tryptophan, 50nM [^2^H_5_][^15^N_2_] indole-3-acetamide, 200nM [^13^C_6_] anthranilate (Supplementary Table S-4). Several solvents, i.e., 80% isopropanol, 100% isopropanol, 50% isopropanol, 80% acetonitrile, and 100% acetonitrile, were tested for optimal extraction recoveries of metabolites from DBS (*n*=3). The optimal extraction solvent was 80% isopropanol (data not shown).

### Protein Extraction and Processing Protocol, and Mass Spectrometry Assays

DBS proteins were extracted, processed, and analyzed by UHPLC–MS as described previously (Vidova et al., 2019). In brief, the DBS extract’s total protein content was determined using BCA (cat. #23227, Thermo Fisher, Waltham, MA) in extracts diluted 100-fold with 50mM ammonium bicarbonate buffer. A dilution series (31.25–2000μg/ml) of bovine serum albumin standard in 50mM ammonium bicarbonate buffer was used to generate a 7-point calibration curve. Spectrophotometric absorbance was measured at 562nm. Mass spectrometry protein assays were performed in 30μl of DBS extract mixed with 10μl of the internal standard solution in 5% of acetonitrile, containing isotopically labeled standard peptides (Supplementary Table S-3) and with 3μl of trypsin (1μg/μl). Samples were incubated (17h at 37°C, orbital shaking), and the enzymatic proteolysis was quenched by adding 200μl of 2% formic acid in water (pH<3). Tryptic peptides were purified and desalted, applying solid phase extraction (Oasis PRIME HLB 96-well plate, 30mg, Waters, Milford, MA). The solid-phase extraction protocol: the sample loaded onto the cartridge, washed with 300μl of 2% formic acid in water (pH<3), eluted with 50% acetonitrile with 2% formic acid (pH<3), and the eluate dried in the SpeedVac. Before UHPLC–MS analysis, peptides were reconstituted in 50μl of 5% acetonitrile with 0.1% formic acid. Processed DBS samples were injected (5μl) on the UHPLC-QQQ system (Infinity 1,260 and 6495B from Agilent Technologies, United States). We utilized a reversed-phase analytical column (C_18_ Peptide CSH; 1.7μm, 2.1mm i.d.×100mm; cat. #186006937, Waters, Milford, MA) and the previously described method (Vidova et al., 2019).

### Mass Spectrometry Metabolite Profiling

Extracted DBS were analyzed in triplicate. Samples were injected (2μl) on the UHPLC-QQQ system equipped with a reverse-phase analytical column (Acquity UHPLC CSH™ C_18_ Column; 1.7μm, 2.1mm x 100mm; cat. #186005297, Waters, Milford, MA) thermostated to 40°C. The mobile phase consisted of buffer A (water with 0.1% formic acid) and buffer B (acetonitrile/water; 95:5 with 0.1% formic acid). The gradient elution program (0–14min) was: 0.0min 5% B, 5min 10% B, 10min 95% B, 11.99 95% B, 12.0 5% B, and 14min 5% B. The mobile phase flow was 0.3ml/min. A standard-flow Jet Stream electrospray source operated in positive SRM ion mode with a capillary voltage of 3.5kV. Additional parameters were: gas flow rate 15l/min at 160°C, sheath gas pressure 25 PSI at 250°C, and nozzle voltage 500V. SRM libraries were generated using Optimizer software (Agilent Technologies) on standard solutions of individual metabolites. For the metabolite identification, 2–4 SRM qualifier transitions were monitored per metabolite (Supplementary Table S-5), and a best-performing SRM transition was used for the quantification (Supplementary Figure S-1). Peak integration and visual inspection were performed in Skyline software (version 20.1.0.155; MacCoss Lab, Univ. of Washington).

### Method Validation

Protein assay validation was reported previously (Vidova et al., 2019). Metabolite profiling assays were validated using matrix-matched calibration curves to determine the linearity range, coefficient of determination (*R*^2^), the limit of detection (LOD), and the limit of quantification (LOQ; Supplementary Figure S-2). LOD and LOQ were established for isotopically labeled standards [^2^H_5_] [^15^N] indole-3-acetamide, [^13^C_6_] indole-3-acetate, [^2^D_4_] L-kynurenine, [^13^C_11_] [^15^N_2_] L-tryptophan, and [^13^C_6_] anthranilic acid in pooled DBS extracts. The dilution series was measured in triplicate. Low concentrations were measured in sextuplicate to determine the standard deviation to establish LOD and LOQ [1] in Supplementary Table S-4. The linearity range was from 1 to 1,200nM for [^2^H_5_] [^15^N] indole-3-acetamide, from 15 to 40,000nM for [^13^C_6_] indole-3-acetate, from 25 to 7,500nM for [^2^D_4_] L-kynurenine, from 7.5 to 75,000nM for [^13^C_11_] [^15^N_2_] L-tryptophan, and from 1 to 880nM for [^13^C_6_] anthranilic acid (Supplementary Table S-6).

### Metabolite Quantification

Concentrations of indole-3-acetamide, indole-3-acetic acid, L-kynurenine, L-tryptophan, and anthranilic acid were determined in DBS extracts using internal standardization with isotopically labeled standards [^2^H_5_] [^15^N] indole-3-acetamide, [^13^C_6_] indole-3-acetate, [^2^D_4_] L-kynurenine), [^13^C_11_] [^15^N_2_] L-tryptophan, and [^13^C_6_] anthranilic acid, at the concentrations of 50, 200, 2000, 20,000, and 200nM, respectively. The calculation uses the concentration of the isotopically labeled standard in the DBS sample and integrated peak areas of the isotopically labeled standard and corresponding metabolite (Equation 1). The concentration of indole-3-aldehyde, indole-3-propionic acid, indole-3-butyric acid, indole-3-lactic acid, and N-acetyl tryptophan was corrected with the response factor (Equation 2), determined in a conventional manner (Vidova and Spacil, 2017; Equation 3).

$$cmet=\left(\frac{CIS}{AIS}xAmet\right)$$(1)

$$\mathrm{cmet}=\left(\frac{CIS}{AIS}xAmet\right):RF$$ (2)

$$RF=\left(\frac{Amet}{AIS}x\frac{CIS}{Cmet}\right)$$(3)

### Statistical Analysis

For metabolites, the median concentration (*n*=3) was used. Metabolite and protein concentrations were log-transformed before statistical analysis, and values below LOQ and LOD were substituted with √2/2 *LOQ and √2/2 *LOD, respectively. Only analytes with <25% substitution were used for the overall statistical analysis as continuous quantitative variables (Antweiler, 2015; Hazra and Gogtay, 2017) Due to a high percentage of values below LOQ, IAM (>68%), IPA (>73%), SAA1(>76%), and CRP (>98%) were used only as additional categorical variables with two categories - “*below LOD*” and “*above LOD*”; the latter further divided into “*below LOD*,” “*below LOQ*,” “*above LOQ*” for visualization purposes.

The Chi-square test was used to test the normality of distributions of logarithmically transformed values. Unpaired one-sided *t* test with Welch correction was used to test significant differences between various groups of samples. The resulting values of *p* were adjusted for multiple hypotheses testing using the Benjamini–Hochberg procedure. Results were considered significant at *FDR*≤0.05. Pearson correlation coefficients (with values of *p* adjusted by Benjamini–Hochberg procedure) were used to describe correlation among metabolites and proteins. Hierarchical clustering with complete-linkage method on Euclidean distance was applied to hierarchically cluster samples (neonates) and distance derived from Pearson correlation to cluster the analytes. Categorical anamnestic data for neonates and their mothers and additional categorical variables (IAM, IPA, SAA1, and CRP) were used to test differences in metabolite and protein levels and correlations between various groups. All statistical analyses were performed in R version 4.0.0 (R core team, 2020) using additional R packages *ggplot2* (Wickham, 2009), *nortest* (Tests for Normality [R package nortest version 1.0-4], 2021; normality testing), *gplots* (Various R Programming Tools for Plotting Data [R package gplots version 3.0.4], 2021), *heatmap3* (Zhao et al., 2014; hierarchal clustering and heat map), *corrplot* (Visualization of a Correlation Matrix [R package corrplot version 0.84], 2021; correlation matrix plot), and *beeswarm* (CRAN, 2021 – Package beeswarm, 9AD; boxplots).

## Results

### Tryptophan and Kynurenine Catabolites and Acute-Phase Proteins in Neonatal Dried Blood Specimens

**Figure 2:**
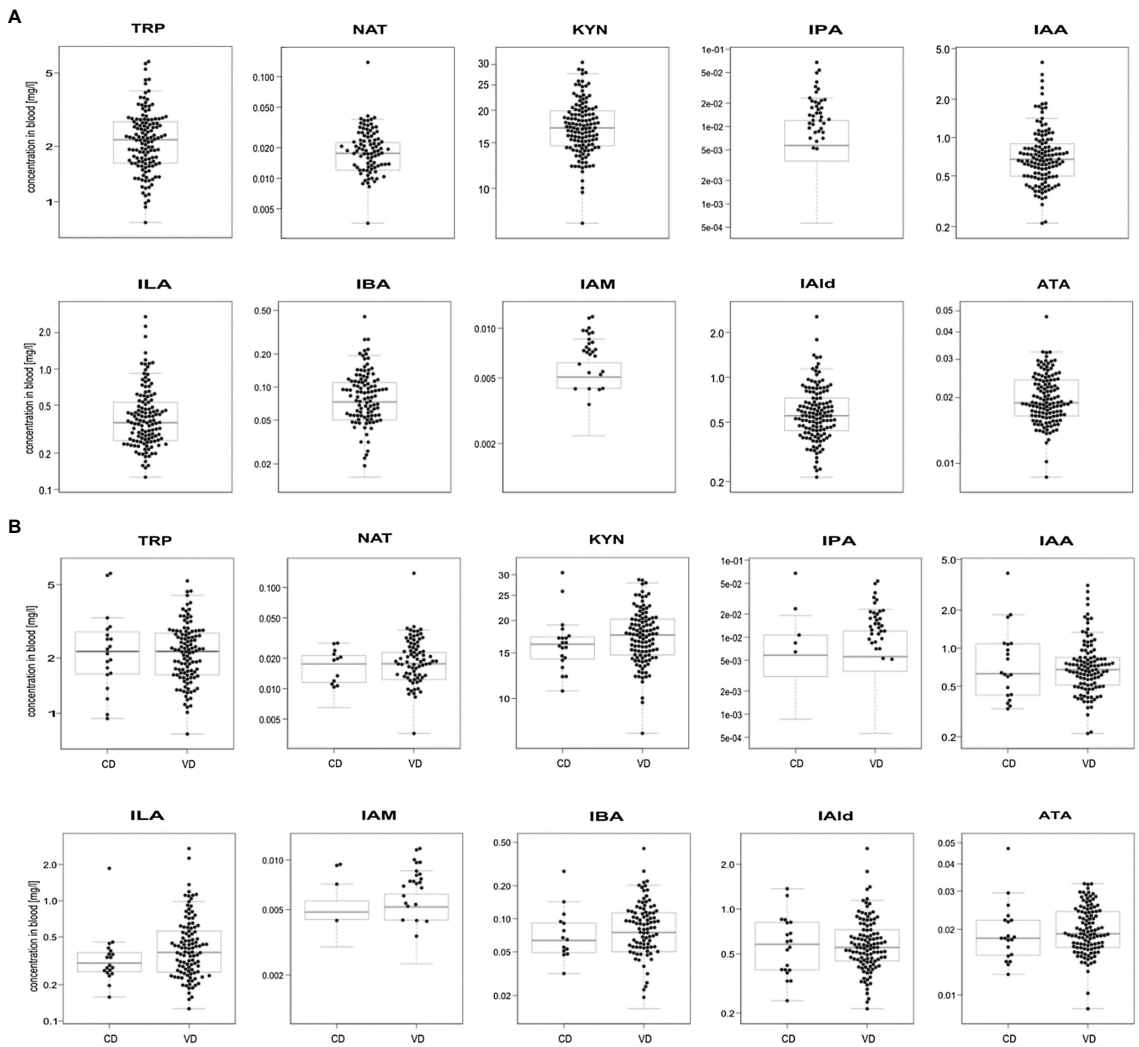
Boxplots of metabolite levels were determined in 134 neonatal DBS **(A)** and grouped according to the delivery mode (VD, vaginal delivery, CD, cesarean delivery; **B**). Sample concentrations >LOQ marked with black dots. Y-axis is in log-scale.

**Figure 3:**
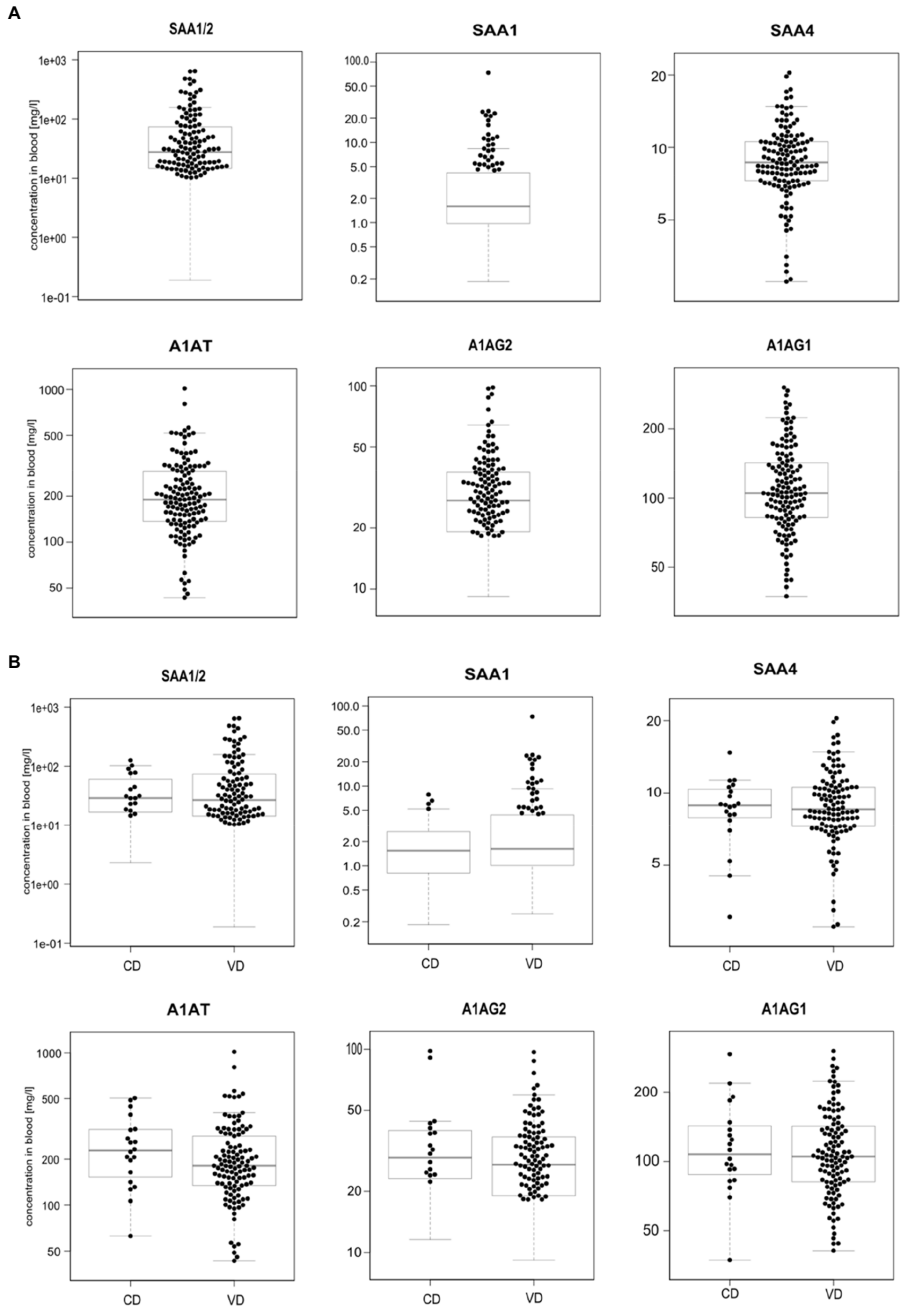
Boxplots of acute-phase protein levels were determined in 134 neonatal DBS **(A)** and grouped according to the delivery mode (VD, vaginal delivery, CD, cesarean delivery; **B**). Sample concentrations >LOQ marked with black dots. Y-axis is in log-scale.

We profiled TRP, ATA, IAA, IAM, IAld, IBA, ILA, IPA, KYN, and NAT levels in 134 neonatal DBS collected on the second day of life (Table 1 and Figure 2A). ATA, IAA, ILA, IPA, KYN, and TRP blood levels were consistent with previous reports in the Human Metabolome Database (2021; HMDB); IAld, IAM, and IBA neonatal levels were newly established (Table 1). IPA, IAM, and SAA1 levels were frequently <LOQ, CRP levels <LOD (see Supplementary Figures S-4, S-5). The median SAA1/2, A1AT, A1AG1, A1AG2, and SAA4 blood levels are in Figure 3A and Table 2. SAA1 blood levels quantified in 33 neonates were elevated (>10mg/l) in 13 VD neonates.

**Table 1:** The lowest, the highest, and median concentrations of tryptophan and kynurenine catabolites in DBS.

Metabolite	Acronym	PubChem CID	# samples >LOQ	Median concentration (mg/L of blood)	The lowest concentration (mg/L of blood)	The highest concentration (mg/L of blood)	HMDB entries (mg/L)	References
Anthranilate	ATA	5,459,842	134	0.0189	0.0086	0.0470	0.0041 +/− 0.0014 [adults (>18years)]	Duranton et al., 2012
Indole-3-acetic acid	IAA	802	134	0.6739	0.2121	3.8892	0.4992 +/− 0.2996 [adults (>18years)]	Duranton et al., 2012
Indole-3-aldehyde	IAld	10,256	134	0.5527	0.2138	2.5499	Expected but not quantified	-
Indole-3-acetamide	IAM	397	42	0.0057	0.0010	0.0341	Expected but not quantified	-
Indole-3-butyric acid	IBA	8,617	96	0.0703	0.0170	0.4377	Detected but not quantified	Cai et al., 2012
Indole-3-lactic acid	ILA	92,904	127	0.3641	0.1262	2.7153	0.57 (0.10–1.03) [adults (>18years)]	Magos, 1987
Indole-3-propionic acid	IPA	3,744	35	0.0072	0.0012	0.0676	0.091 (0.055–0.21) [adults (>18years)]	Danaceau et al., 2003
Kynurenine	KYN	846	134	17.0882	7.3402	30.5551	1.23 (1.02–1.47) [neonates (0–30days)]	Herberth et al., 2015
N-Acetyltryptophan	NAT	700,653	108	0.0173	0.0030	0.1382	Not quantified in blood	-
Tryptophan	TRP	6,305	134	2.1699	0.7715	5.7512	3.676–11.23 [neonates (0–30days)]	Human Metabolome Database, 2021

**Table 2:** The lowest, the highest, and median levels of acute-phase proteins in DBS.

Protein	UniProt entry	Surrogate peptide	# samples >LOQ	Median concentration (mg/L of blood)	The lowest concentration (mg/L of blood)	The highest concentration (mg/L of blood)
SAA1	**PODJI8**	**FFGHGAEDSLADQAANEWGR**	33	8.1	<LOQ	73.6
SAA1/2	**PODJI8/PODJI9**	**SFFSFLGEAFDGAR**	120	30.9	<LOQ	644.9
SAA4	**P35542**	**FRPDGLPK**	134	8.7	2.8	20.4
CRP	**P02741**	**ESDTSYVSLK**	2	N/A	<LOQ	7.2
A1AT-1	**P01009-1**	**AVLTIDEK**	134	189.8	43.2	1016.1
A1AG1	**P02763**	**NWGLSVYADKPETTK**	134	105.0	37.3	302.7
A1AG2	**P19652**	**SDVMYTDWK**	107	32.0	<LOQ	98.0

No significant differences were observed in metabolite (Figure 2B) and APP levels (Figure 3B) between CD (*n*=20) and VD (*n*=114) groups and respective to clinical conditions in mothers and neonates (Supplementary Figures S-4, S-5). SAA1/2, SAA4, and A1AG1 showed statistically significantly higher levels (*p*<0.0001) in sample groups with CRP>LOD compared to samples with CRP<LOD. The same difference was observed in the sample groups with SAA1 above and below LOD (Supplementary Figure S-6). The heatmap of protein and metabolite concentrations with rows and columns ordered based on unsupervised clustering of the analytes (Pearson correlation-based distance, main clusters A, B, C) and the DBS samples (Euclidean distance, main clusters D, E) is shown in Supplementary Figure S-4. Cluster A represents APPs (however, A1AT shows a weak correlation with other APPs), IBA, IAA, and IAld fall into cluster B, and cluster C consists of KYN, ATA, TRP, NAT, and ILA. Clusters D and E split DBS samples into two different groups – cluster E is characterized primarily with higher APP (except A1AT) and metabolites levels. Mothers’ and neonates’ anamnestic data added into the picture show no parameter related to clusters D or E. Additional categorical variables CRP, SAA1, IPA, and IAM showed in the figure indicate that cluster E is connected with higher CRP and SAA1 levels. Similar and even more apparent trends in neonates’ clustering are visible in cluster analysis based on proteins only (Supplementary Figure S-5). The APPs SAA1/2, SAA1, CRP, A1AG1, and A1AG2 are elevated in the blood (cluster D in Supplementary Figure S-5). High A1AT levels are observed both in cluster D and cluster E (Supplementary Figure S-5). In cluster E, there are low levels of other APPs. Elevated A1AT levels are caused by infection and also contraception, pregnancy, thyroid infection, or stress. In neonates, increased A1AT levels in cluster E can be associated with stress factors acting during delivery. However, neonates’ anamnestic data did not show any relation to the clusters.

### The Correlation Between Metabolite and Acute-Phase Protein Blood Levels

**Figure 4:**
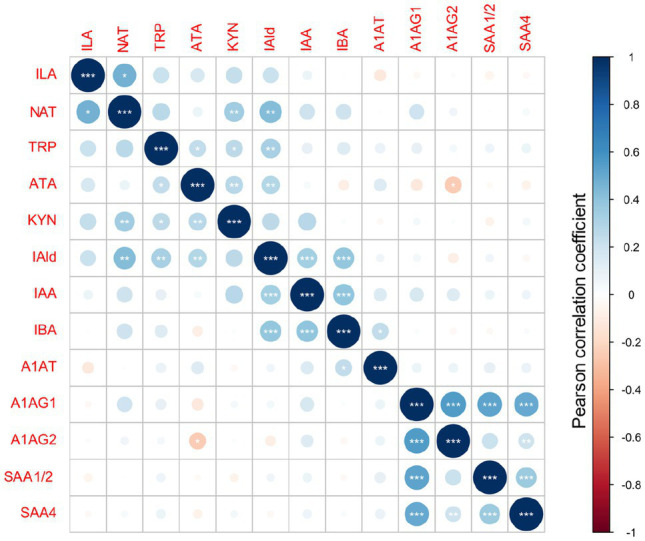
Metabolites and acute-phase proteins correlation in DBS. Values of Pearson correlation coefficients are color-coded. The statistical significance is marked with *** for *p*<0.001, ** for *p*<0.01 and * for *p*<0.05.

**Figure 5:**
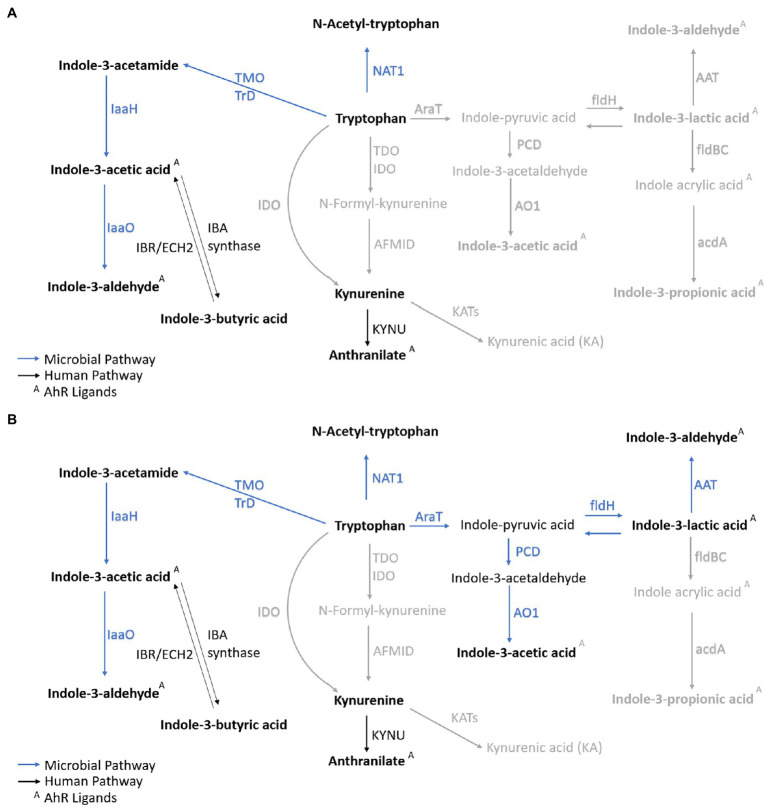
Metabolic profile of VD and CD neonates. Correlated metabolites (*p*<0.01 and/or *p*<0.001) in VD neonates **(A)** and CD neonates **(B)** are marked in bold. Nonsignificant correlations are marked in gray. The microbial catabolites with significant correlations are highlighted in dark blue. The host metabolism products are marked in black. ArAT, aromatic amino acid aminotransferase, AAT, aromatic amino acid transferase, acdA, acyl-CoA dehydrogenase, AFMID, kynurenine formamidase, AO1, indole acetaldehyde oxidase, ECH2, enoyl-CoA hydratase-2, fldH, phenyl lactate dehydrogenase, fldBC, phenyl lactate dehydratase, IDO, indoleamine-2,3-dioxygenase, IaaH, indole acetamide hydrolase, IaaO, indole-3-acetic acid oxidase, KYNU, kynureninase, KAT, kynurenine amino transferase, MAO, monoamine oxygenase, NAT1, arylamine N-acetyltransferase 1, porB,C, pyruvate ferredoxin oxidoreductase B and C, PCD, pyruvate decarboxylase, TDO, tryptophan-2,3-dioxygenase, TMO, tryptophan-2-monooxygenase, TrD, tryptophan decarboxylase.

The overall metabolites and proteins correlation matrix plot is shown in Figure 4. A negative Pearson’s correlation (*p*<0.05) was observed for ATA/A1AG2 pair, and a positive Pearson correlation (*p*<0.05) was between the A1AT/IBA pair. Metabolite precursor and product pairs were correlated (*p*<0.001) – for instance, IAld/IAA, IAld/IBA, and IBA/IAA (Figures 1B, 4, 5). Significant correlations (*p*<0.01 and *p*<0.001) for metabolite pairs were observed for VD neonates (Supplementary Figure S-7) and all 134 neonates (Figure 4).

The VD subgroup (Supplementary Figure S-7) showed positive statistically significant correlations between metabolites and nonsignificant correlations between metabolites and proteins. The correlation pattern between metabolites and proteins in the CD subgroup differed from VD neonates (Supplementary Figure S-7). Positively correlated were A1AG1/SAA4 proteins (*p*<0.01) and the A1AG1/A1AG2 isoforms (*p*<0.001). In contrast with the VD subgroup, putative negative correlations were noted in the CD subgroup for IAA/NAT, IAA/ATA, TRP/IAA, IBA/ATA, ATA/(A1AG1 and A1AG2), IAld/SAA4, and ILA/A1AT pairs (Supplementary Figure S-7). Several metabolite/metabolite and metabolite/protein pairs showed a reversed correlation (positive/negative) comparing VD and CD groups. For instance, the ILA/IAA pair positively correlated in VD but negatively in CD neonates (Supplementary Figure S-8).

Different enzymes and, therefore, pathways are enriched depending on the delivery mode. The most significant correlations indicate metabolites converted from TRP *via* microbial TMO/TrD in VD neonates (Figures 4, 5A). For CD neonates, the enriched enzymes are TMO/TrD and AAT, fldH, and AO1, producing ILA, IAld, and IAA (Figure 5B).

## Discussion

This study aimed to investigate immunomodulatory microbial tryptophan and kynurenine ligands to AHR and PXR along with APP levels in neonatal DBS to explore potential correlations or patterns specific to the delivery mode (CS and VD). We developed a protocol for simultaneous APP quantification and microbial catabolites profiling in neonatal DBS. IAA levels profiled in DBS were reported previously (Dénes et al., 2012; Freeman et al., 2018). However, we are the first to report neonatal IAld, IBA, and IAM levels.

### The Mode of Delivery, Circulating Metabolite Profile, and Protein Levels

The circulating profile of tryptophan and kynurenine catabolites and APPs was compared in CD (*n*=20) and VD (*n*=114) neonates. Microbial enzymes (i.e., TMO/TrD, NAT1, IaaH, and IaaO) characterize VD neonates’ metabolic profiles. On the other hand, AraT, fldH, AAT, and AO1 primarily determined the CD neonates’ metabolite profile. High SAA1 levels (>10mg/l) were observed in 13 VD neonates. However, the overall difference between VD and CD groups was not statistically significant. The microbial colonization and immune system response develop rapidly on the second day of life (Martin et al., 2010). Shao et al. compared the microbial diversity in CD and VD neonates and found substantial differences between the first and the fourth day of life (Shao et al., 2019).

### Supportive Role of Acute-Phase Proteins to the Immune System

We attempted to link metabolite and protein profiles in DBS to clinical anamnestic data. Each APP has a unique role in shaping an infant’s immune system, and there is a cross-talk between quantified metabolites and APPs. Once the inflammation signal passes through IL-6 and IL-1 to APP production in hepatocytes, these proteins trigger a systemic response to modulate the immune system (Ackermann, 2017; Zachary, 2017). A1AT inhibits the production of TNF-α and the metalloprotease in macrophages and regulates CD14 and TLR4 expression to reduce pro-inflammatory stimuli (i.e., IL-1 and IL-6) and upregulate anti-inflammatory cytokines (i.e., IL-10, TGF-ß; Breit et al., 1985; Bergin et al., 2012; Baraldo et al., 2015; Cosio et al., 2016). In murine and human studies, A1AT modulates dendritic cells and increases FoxP3^+^ T-regulatory cells (Marcondes et al., 2014; Berger et al., 2018). Inflammatory cytokines (i.e., IL-6, IL-1, and TNF) primarily regulate APPs production in hepatocytes (Ackermann, 2017; Zachary, 2017). Pro-inflammatory cytokines stimulate an essential IDO1 pathway in macrophages (Alberati-Giani et al., 1996; Prendergast et al., 2011). TRP and KYN are the rate-limiting substrates for the IDO1 enzyme (Zhang et al., 2019).

KYN pathway is one of the main degradation routes for dietary tryptophan (Figure 1B). IDO converts TRP to KYN, a crucial metabolite in maintaining immune homeostasis (Ding et al., 2020; Wyatt and Greathouse, 2021). In humans is encoded by the IDO1 gene expressed in immune cells (i.e., monocytes, macrophages, and dendritic cells), necessary in antigen presentation (Nikolaus et al., 2017). As investigated in this study, IDO expression regulates T-cell differentiation to avoid tissue damage and oxidative stress (Le Floc’h et al., 2011). KYN metabolites can cross the blood–brain barrier further and act as neuroprotectants (Roth et al., 2021; Wyatt and Greathouse, 2021). KYN enters the brain from the blood circulation *via* the amino acid transporter, taken up by astrocytes and microglial cells (Atilla and Basak, 2015).

### The Interaction Between Catabolites of Tryptophan and Acute-Phase Proteins

TRP, an essential amino acid in human nutrition, cannot be produced in mammalian cells (Atilla and Basak, 2015). The indole catabolites of TRP mediate the immune system development and homeostasis *via* various mechanisms of action. Our results suggest a cross-talk between metabolites and APPs observed as diverse correlations between metabolites and APPs relative to the mode of delivery. For instance, a stronger negative correlation between A1AG2/ATA was observed in CD compared to VD neonates. The APP and metabolite pair, A1AG2 and ATA, both carry out supportive functions essential for developing the neonatal immune system. The metabolites such as ATA and KYN have an epigenetic effect in the methylation and glycosylation of hypothalamic neuronal peptide coding genes and neuronal differentiation-related loci (increase in H3K4 methylation and H2AS40 O-GlcNAcylation). By increasing methylation and histone modification, gene expression is stabilized, and DNA mutation is avoided (Hayakawa et al., 2019).

TRP catabolites are AhR and/or PXR ligands assuring in their function the healthy development of the neonate (Li et al., 2021). The paramount importance is establishing the gut barrier and blood–brain barrier (BBB) function in early life development. The aryl hydrocarbon receptor is a ligand-receptor transcription factor (TF) activated by TRP and its metabolites. The TF is expressed by many immune system cells such as macrophages, dendritic cells, NK cells, B lymphocytes, and subtypes of T cells as Th17 and Treg cells (Ambrosio et al., 2019). PXR is also expressed in many cells, most widely in the liver, intestines, kidneys, and intestinal epithelial cells. The ligand-activated TF is activated by naturally occurring steroids and synthetic glucocorticoids. Furthermore, the PXR receptor controls various physiological processes and the metabolism of lipids, glucose, and bile acids (Illés et al., 2020).

IPA was shown to fortify the intestinal barrier by engaging the PXR. IPA is produced by gut microbiota from dietary TRP, which accumulates the host serum (Danaceau et al., 2003; Dodd et al., 2017). IPA activates PXR and induces downregulation of the toll-like receptors, mainly TLR4, and its downstream signaling pathway. In the murine intestine, IPA downregulated enterocytes-mediated inflammatory cytokine TNFα and upregulated junctional protein markers (Venkatesh et al., 2014). The essential gene for IPA, synthesized by aromatic amino acid metabolism in the gut by the bacterium *Clostridium sporogenes*, is *fldC* with a broad impact on human immune cells (Dodd et al., 2017). The authors observed a different spectrum of adaptive immune response in Δ*fldC* mutant. The *fldC* mutant showed higher circulating myeloid cells, including neutrophils and Ly6C^+^ monocytes and increased antigen-experienced effector/memory T cells. In addition, secretory IgA levels were increased in *fldC* mutant mice (Dodd et al., 2017). IPA plays an essential role in intestinal barrier regulation, also crucial in the physiological development of neonates after delivery.

IPA has further also radical scavenging activity and has neuronal properties (Kaufmann, 2018). It inhibits β-amyloid fibril formation and can act as a neuroprotectant against various oxidants (Bendheim et al., 2002). IPA also has chemical chaperone activity and suppresses endoplasmic reticulum stress-induced neuronal cell death (Mimori et al., 2019). Further PXR agonists are IAM and IAA. This interaction through the PXR leads to the inhibition of NF-*κ*B signaling pathway (Illés et al., 2020). Therefore, PXR has anti-inflammatory properties (Zhou et al., 2006; Okamura et al., 2020).

We observed significant correlations indicating metabolites conversion from TRP to NAT, IAld, KYN, and ATA *via* microbial TMO/TrD and NAT in VD neonates. The profiled metabolites show the importance of a gut–brain axis in the systemic response and intestinal homeostasis regulation. For example, NAT is a substance P-receptor antagonist (Fernandes et al., 2018) and a neuroprotective agent (Sirianni et al., 2015). IAld, as an AHR agonist, stimulates the production of IL-22 (Zelante et al., 2013). The cytokine IL-22, produced in the liver, kidneys, pancreas, skin, and intestine, induces tissue regeneration and supports antimicrobial molecules’ production, helping develop a defense line against tissue damage and microbial infection (Dudakov et al., 2015). The mucosal immune homeostasis was recently investigated in a murine model of autoimmune inflammation. IAld administered in the gut alleviated hepatic inflammation and fibrosis by modulating the intestinal microbiota by activating the AhR-IL-22-axis to restore mucosal integrity (D’Onofrio et al., 2021). It agrees with the finding that microbial-produced IAld further provides mucosal protection from inflammation in the host innate immune system, where the cytokine IL-22 *via* AhR receptor promoted IL-18 expression. Both the innate and the adaptive immune system are involved (Borghi et al., 2019). Furthermore, IAld attenuates the increase in epithelial permeability caused by stimulation with a pro-inflammatory cytokine TNFα in a dose-dependent manner (Scott et al., 2020). IAld regulates gut barrier integrity through tight junctions (e.g., zonulin and occludin) and adherens junctions, which are essential for regulating intestinal permeability (Zihni et al., 2016; Scott et al., 2020).

For CD neonates, the enriched enzymes are TMO/TrD and AAT, fldH, and AO1, producing KYN, ILA, IAld, and IAA. The different enriched enzymes and pathways show that other routes are taken on the second day of life in CD and VD neonates, visible and emphasized in the correlation matrix plots for VD and CD neonates, showing markedly different patterns. The function of ILA was investigated in the gnotobiotic mice model, and it was found that ILA reprograms intraepithelial lymphocytes (IELs, CD4^+^T cells) into double-positive IELs (CD8aa^+^CD4^+^) with immunoregulatory function (Cervantes-Barragan et al., 2017). Moreover, ILA from breastmilk was identified as an anti-inflammatory metabolite. ILA requires the interaction with TLR4 and the AHR receptor to interfere with its transcription of the inflammatory cytokine IL-8 that causes excessive inflammation in the premature intestine (Meng et al., 2020). *In vivo* and *in vitro* results showed pleiotropic protective effects on immature enterocytes, including anti-inflammatory, antiviral, and developmental regulatory potential in a region-dependent and age-dependent manner. The further transcriptomic analysis showed that ILA has a regulatory effect on the STAT1 pathway. The STAT1 pathway plays an essential role in IL-1β-induced inflammation (Huang et al., 2021).

ATA is a product of dietary tryptophan and has anti-inflammatory properties on Na^+^/dicarboxylate cotransporters, NaDC1, and NaCT (Pajor and Sun, 2013). IAA levels positively correlate with intestinal IL-22 levels, through which antimicrobial proteins are targeted, and mucosal inflammation is downregulated (Laurans et al., 2018; Natividad et al., 2018). In addition, IAA has a protective effect against lipopolysaccharide (LPS)-induced inflammatory response and free radical generation in macrophages. IAA significantly ameliorated LPS-induced expression of interleukin-1β (IL-1β), interleukin-6 (IL-6), and monocyte chemoattractant protein-1 (MCP-1) as well as generation of reactive oxidative species (ROS) and nitric oxide (NO). LPS-triggered nuclear translocation of nuclear factor kappa B (NF-κB) p65 was mitigated by IAA treatment (Ji et al., 2020). In a previous study, Ji et al. showed in mice that IAA mitigates high-fat diet-induced evaluation in fasting blood glucose and total plasma cholesterol, low-density lipoprotein cholesterol, and glutamic pyruvic transaminase activity. IAA supports the liver function linked with mitigated total triglycerides and cholesterol concentration and upregulation of genes involved in lipogenesis. Furthermore, IAA was shown to protect against reactive oxygen species and attenuate the inflammatory response in the liver of mice exposed to a high-fat diet (Ji et al., 2019).

Overall, we profiled microbial metabolites of the kynurenine and tryptophan pathway and acute-phase proteins in 3μl of dried blood, and we first reported neonatal IAld, IBA, and IAM levels. We observed divergent metabolic profiles in VD and CD neonates. The different colonization of the initial microbial metabolites could be caused by distinct microbial tryptophan degradation routes in VD and CD. In VD, the enriched pathways could lead to higher NAT, KYN, and ATA metabolite levels. In CD neonates, the enriched bacterial enzymes could lead to higher KYN, IAld, ILA, and IAA levels. Despite the diverse TRP catabolism, our results point to indole catabolites’ distinct profile on the second day of life in VD and CD neonates, demonstrated through different TRP metabolites, independently of unique postnatal microbial colonization at VD and CD (Sanidad and Zeng, 2020).

We quantified in our study indole catabolites in both delivery modalities (vaginal and cesarean delivery) but found no significant differences in both groups. However, this study’s limitations are the small number of CD neonates relative to VD and a single-point sampling. Correlations between metabolites and proteins in CD neonates require validation in a more extensive follow-up cohort study. In summary, we attempted to elucidate the mechanism of the immunomodulatory function of microbial metabolites. A further potential distinction will develop in the infant’s microbiome composition and metabolite profile over time. Our findings suggest the supportive role of human gut microbiota in developing and maintaining immune system homeostasis.

## Data Availability Statement

The datasets presented in this study can be found in online repositories at https://panoramaweb.org/Aust_et_al_SI.url and the ProteomeXchange ID: PXD027606.

## Ethics Statement

The studies involving human participants were reviewed and approved by the Committee for Ethics of CELSPAC: TNG (CELSPAC/EK/4/2016) at University Hospital Brno, Czechia. Written informed consent to participate in this study was provided by the participants’ legal guardian/next of kin.

## Author Contributions

A-CA, EBe, KC, VV, and ZS designed the experiments. A-CA, KC, and EBe carried out the experiments and analyzed the data. IB and PJ recruited the study subjects and collected the biological samples. SS and EBu performed the statistical analysis. A-CA, EBe, VT, and ZS wrote the manuscript with input from all authors. JK, VT, and ZS conceived the study and supervised the project. All authors contributed to the article and approved the submitted version.

## Funding

The study was supported by the Grant Agency of the Czech Republic (17-24592Y), the RECETOX research infrastructure (LM2018121) financed by the Ministry of Education, Youth and Sports, and Operational Programme Research, Development, and Innovation - project CETOCOEN EXCELLENCE (CZ.02.1.01/0.0/0.0/17_043/0009632) and project CETOCOEN PLUS (CZ.02.1.01/0.0/0.0/15_003/0000469) and the Ministry of Health, the Czech Republic – FNBr (65269705).

## Conflict of Interest

The authors declare that the research was conducted in the absence of any commercial or financial relationships that could be construed as a potential conflict of interest.

## Publisher’s Note

All claims expressed in this article are solely those of the authors and do not necessarily represent those of their affiliated organizations, or those of the publisher, the editors and the reviewers. Any product that may be evaluated in this article, or claim that may be made by its manufacturer, is not guaranteed or endorsed by the publisher.

## Abbreviations

A1AT: Alpha-1-antitrypsin
A1AG1: Alpha-1-acid glycoprotein 1
A1AG2: Alpha-1-acid glycoprotein 2
AAT: Aromatic amino acid transferase
acdA: Acyl-CoA dehydrogenase
AFMID: Kynurenine formamidase
AHR: Aryl-hydrocarbon receptor
AO1: Indole acetaldehyde oxidase
APP: Acute-phase protein
ArAT: Aromatic amino acid aminotransferase
ATA: Anthranilate
BCA: Bicinchoninic acid
CD: Cesarean delivery
CELSPAC-TNG: Central European Longitudinal Studies of Parents and Children – The Next Generation
CRP: C-reactive protein
DBS: Dried blood specimens
ECH2: Enoyl-CoA hydratase 2
fldH: Phenyl lactate dehydrogenase
fldBC: Phenyl lactate dehydratase
FoxP3: Forkhead-box-protein 3
HIAA: Hydroxy-indole acetic acid
HMDB: Human Metabolome Database
IAA: Indole-3-acetic acid
IaaH: Indole acetamide hydrolase
IaaO: Indole-3-acetic acid oxidase
IAld: Indole-3-aldehyde
IAM: Indole-3-acetamide
IBA: Indole-3-butyric acid
IBS: Inflammatory bowel syndrome
IDO: Indoleamine-2,3-dioxygenase
ILA: Indole-3-lactic acid
IL-1: Interleukin-1
IL-2: Interleukin-2
IL-6: Interleukin-6
IL-8: Interleukin-8
IL-10: Interleukin 10
IL-22: Interleukin 22
ILC3: Innate lymphoid cells 3
INFγ: Interferon-γ
IPA: Indole-3-propionic acid
KAT: Kynurenine amino transferase
KYN: Kynurenine
KYNU: Kynureninase
LC: Liquid chromatography
MAO: Monoamine oxygenase
MS: Mass spectrometry
NAT: N-acetyl-tryptophan
NAT1: Arylamine N-acetyltransferase 1
PCD: Pyruvate decarboxylase
porB/C: Pyruvate ferredoxin oxidoreductase B and C
PXR: Pregnane X receptor
SAA: Serum amyloid A
SRM: Selected reaction monitoring
TDO: Tryptophan-2,3-dioxygenase
TGF β: Transforming growth factor
TH1: t helper type 1 cells
TH2: t helper type 2 cells
TLR4: Toll like receptor 4
TMO: tryptophan-2-monooxygenase
TNFα: Tumor necrosis factor α
TrD: Tryptophan decarboxylase
TRP: Tryptophan
UHPLC: Ultra-high-performance liquid chromatography
VD: Vaginal delivery
QQQ: Triple quadrupole

## References

[ref1] Ackermann M. R. (2017). “Chapter 3: Inflammation and healing,” in Pathologic Basis of Veterinary Disease. 6th Edn. ed. Zachary J. F. (Amsterdam, Netherlands: Mosby), 73.e2–131.e2.

[ref2] Agus A. Planchais J. Sokol H. (2018). Gut microbiota regulation of tryptophan metabolism in health and disease. Cell Host Microbe 23, 716–724. doi: 10.1016/j.chom.2018.05.003, PMID: 29902437

[ref3] Alberati-Giani D. Ricciardi-Castagnoli P. Köhler C. Cesura A. M. (1996). Regulation of the kynurenine metabolic pathway by interferon-gamma in murine cloned macrophages and microglial cells. J. Neurochem. 66, 996–1004. doi: 10.1046/j.1471-4159.1996.66030996.x, PMID: 8769859

[ref4] Alexeev E. E. Lanis J. M. Schwisow K. D. Kominsky D. J. Colgan S. P. (2016). Microbiota-derived tryptophan metabolites activate aryl hydrocarbon receptor and induce IL-10 receptor expression in intestinal epithelia. FASEB J. 30:57.2. doi: 10.1096/fasebj.30.1_supplement.57.2

[ref5] Ambrosio L. F. Insfran C. Volpini X. Rodriguez E. A. Serra H. M. Quintana F. J.. (2019). Role of aryl hydrocarbon receptor (AhR) in the regulation of immunity and immunopathology during trypanosoma cruzi infection. Front. Immunol. 10:631. doi: 10.3389/fimmu.2019.00631, PMID: 30984194 PMC6450169

[ref6] Antweiler R. C. (2015). Evaluation of statistical treatments of left-censored environmental data using coincident uncensored data sets. II. Group comparisons. Environ. Sci. Technol. 49, 13439–13446. doi: 10.1021/acs.est.5b02385, PMID: 26490190

[ref7] Aragozzini F. Ferrari A. Pacini N. Gualandris R. (1979). Indole-3-lactic acid as a tryptophan metabolite produced by *Bifidobacterium* spp. Appl. Environ. Microbiol. 38, 544–546. doi: 10.1128/aem.38.3.544-546.1979, PMID: 533277 PMC243529

[ref8] Arnon S. Litmanovitz I. Regev R. H. Bauer S. Shainkin-Kestenbaum R. Dolfin T. (2007). Serum amyloid A: an early and accurate marker of neonatal early-onset sepsis. J. Perinatol. 27, 297–302. doi: 10.1038/sj.jp.7211682, PMID: 17344924

[ref9] Atilla E. Basak E. A. (2015). Tryptophan Metabolism: Implications for Biological Processes, Health and Disease. 1st Edn. eds. Engin A. Engin A. B. (Totowa, New Jersey, United States: Humana Press).

[ref10] Baraldo S. Turato G. Lunardi F. Bazzan E. Schiavon M. Ferrarotti I.. (2015). Immune activation in α1-antitrypsin-deficiency emphysema. Beyond the protease-antiprotease paradigm. Am. J. Respir. Crit. Care Med. 191, 402–409. doi: 10.1164/rccm.201403-0529OC, PMID: 25412116

[ref11] Bendheim P. E. Poeggeler B. Neria E. Ziv V. Pappolla M. A. Chain D. G. (2002). Development of indole-3-propionic acid (OXIGON™) for Alzheimer’s disease. J. Mol. Neurosci. 19, 213–217. doi: 10.1007/s12031-002-0036-0, PMID: 12212784

[ref12] Bennet R. Nord C. E. (1987). Development of the faecal anaerobic microflora after caesarean section and treatment with antibiotics in newborn infants. Infection 15, 332–336. doi: 10.1007/BF01647733, PMID: 3692604

[ref13] Berger M. Liu M. Uknis M. E. Koulmanda M. (2018). Alpha-1-antitrypsin in cell and organ transplantation. Am. J. Transplant. 18, 1589–1595. doi: 10.1111/ajt.14756, PMID: 29607607 PMC6055806

[ref14] Bergin D. A. Hurley K. McElvaney N. G. Reeves E. P. (2012). Alpha-1 antitrypsin: a potent anti-inflammatory and potential novel therapeutic agent. Arch. Immunol. Ther. Exp. 60, 81–97. doi: 10.1007/s00005-012-0162-5, PMID: 22349104

[ref15] Bezirtzoglou E. (1997). The intestinal microflora during the first weeks of life. Anaerobe 3, 173–177. doi: 10.1006/anae.1997.0102, PMID: 16887585

[ref16] Borghi M. Pariano M. Solito V. Puccetti M. Bellet M. M. Stincardini C.. (2019). Targeting the aryl hydrocarbon receptor with indole-3-aldehyde protects from vulvovaginal candidiasis via the IL-22-IL-18 cross-talk. Front. Immunol. 10:2364. doi: 10.3389/fimmu.2019.02364, PMID: 31681274 PMC6798081

[ref17] Breit S. N. Wakefield D. Robinson J. P. Luckhurst E. Clark P. Penny R. (1985). The role of alpha 1-antitrypsin deficiency in the pathogenesis of immune disorders. Clin. Immunol. Immunopathol. 35, 363–380. doi: 10.1016/0090-1229(85)90097-2, PMID: 3886224

[ref18] Buck M. Gouwy M. Wang J. Snick J. Opdenakker G. Struyf S.. (2016). Structure and expression of different serum amyloid A (SAA) variants and their concentration-dependent functions during host insults. Curr. Med. Chem. 23, 1725–1755. doi: 10.2174/0929867323666160418114600, PMID: 27087246 PMC5405626

[ref19] Cai H.-L. Li H.-D. Yan X.-Z. Sun B. Zhang Q. Yan M.. (2012). Metabolomic analysis of biochemical changes in the plasma and urine of first-episode neuroleptic-naïve schizophrenia patients after treatment with risperidone. J. Proteome Res. 11, 4338–4350. doi: 10.1021/pr300459d, PMID: 22800120

[ref20] Cervantes-Barragan L. Chai J. N. Tianero M. D. Di Luccia B. Ahern P. P. Merriman J.. (2017). *Lactobacillus reuteri* induces gut intraepithelial CD4+CD8αα+ T cells. Science 357, 806–810. doi: 10.1126/science.aah5825, PMID: 28775213 PMC5687812

[ref21] Clyne B. Olshaker J. S. (1999). The C-reactive protein. J. Emerg. Med. 17, 1019–1025. doi: 10.1016/S0736-4679(99)00135-3, PMID: 10595891

[ref22] Cosio M. G. Bazzan E. Rigobello C. Tinè M. Turato G. Baraldo S.. (2016). Alpha-1 antitrypsin deficiency: beyond the protease/antiprotease paradigm. Ann. Am. Thorac. Soc. 13(Suppl. 4), S305–S310. doi: 10.1513/AnnalsATS.201510-671KV, PMID: 27564665

[ref23] CRAN (2021). Package beeswarm (9AD). Available at: https://cran.r-project.org/web/packages/beeswarm/index.html (Accessed August 6, 2021).

[ref25] Danaceau J. P. Anderson G. M. McMahon W. M. Crouch D. J. (2003). A liquid chromatographic-tandem mass spectrometric method for the analysis of serotonin and related indoles in human whole blood. J. Anal. Toxicol. 27, 440–444. doi: 10.1093/jat/27.7.440, PMID: 14606996

[ref26] Dénes J. Szabó E. Robinette S. L. Szatmári I. Szőnyi L. Kreuder J. G.. (2012). Metabonomics of newborn screening dried blood spot samples: a novel approach in the screening and diagnostics of inborn errors of metabolism. Anal. Chem. 84, 10113–10120. doi: 10.1021/ac302527m, PMID: 23094949

[ref27] Dileep K. V. Remya C. Tintu I. Haridas M. Sadasivan C. (2013). Interactions of selected indole derivatives with phospholipase A₂: *in silico* and *in vitro* analysis. J. Mol. Model. 19, 1811–1817. doi: 10.1007/s00894-012-1741-4, PMID: 23315198

[ref28] Ding X. Bin P. Wu W. Chang Y. Zhu G. (2020). Tryptophan metabolism, regulatory T cells, and inflammatory bowel disease: a mini review. Mediat. Inflamm. 2020:9706140. doi: 10.1155/2020/9706140, PMID: 32617076 PMC7306093

[ref29] Dodd D. Spitzer M. H. Van Treuren W. Merrill B. D. Hryckowian A. J. Higginbottom S. K.. (2017). A gut bacterial pathway metabolizes aromatic amino acids into nine circulating metabolites. Nature 551, 648–652. doi: 10.1038/nature24661, PMID: 29168502 PMC5850949

[ref24] D’Onofrio F. Renga G. Puccetti M. Pariano M. Bellet M. M. Santarelli I.. (2021). Indole-3-carboxaldehyde restores gut mucosal integrity and protects from liver fibrosis in murine sclerosing cholangitis. Cell 10:1622. doi: 10.3390/cells10071622, PMID: 34209524 PMC8305598

[ref30] Dudakov J. A. Hanash A. M. van den Brink M. R. M. (2015). Interleukin-22: immunobiology and pathology. Annu. Rev. Immunol. 33, 747–785. doi: 10.1146/annurev-immunol-032414-112123, PMID: 25706098 PMC4407497

[ref31] Duranton F. Cohen G. Smet R. Rodriguez M. Jankowski J. Vanholder R.. (2012). Normal and pathologic concentrations of uremic toxins. J. Am. Soc. Nephrol. 23, 1258–1270. doi: 10.1681/ASN.2011121175, PMID: 22626821 PMC3380651

[ref32] Elsden S. R. Hilton M. G. Waller J. M. (1976). The end products of the metabolism of aromatic amino acids by *Clostridia*. Arch. Microbiol. 107, 283–288. doi: 10.1007/BF00425340, PMID: 1275638

[ref33] Fernandes J. Mudgal J. Rao C. M. Arora D. Basu Mallik S. Pai K. S. R.. (2018). N-acetyl-L-tryptophan, a substance-P receptor antagonist attenuates aluminum-induced spatial memory deficit in rats. Toxicol. Mech. Methods 28, 328–334. doi: 10.1080/15376516.2017.1411412, PMID: 29185389

[ref34] Finegold S. M. Molitoris D. Song Y. Liu C. Vaisanen M.-L. Bolte E.. (2002). Gastrointestinal microflora studies in late-onset autism. Clin. Infect. Dis. 35, S6–S16. doi: 10.1086/341914, PMID: 12173102

[ref35] Francino M. P. (2018). Birth mode-related differences in gut microbiota colonization and immune system development. Ann. Nutr. Metab. 73(Suppl. 3), 12–16. doi: 10.1159/000490842, PMID: 30041189

[ref36] Freeman J. D. Rosman L. M. Ratcliff J. D. Strickland P. T. Graham D. R. Silbergeld E. K. (2018). State of the science in dried blood spots. Clin. Chem. 64, 656–679. doi: 10.1373/clinchem.2017.275966, PMID: 29187355

[ref37] Gao J. Xu K. Liu H. Liu G. Bai M. Peng C.. (2018). Impact of the gut microbiota on intestinal immunity mediated by tryptophan metabolism. Front. Cell. Infect. Microbiol. 8:13. doi: 10.3389/fcimb.2018.00013, PMID: 29468141 PMC5808205

[ref38] Grönlund M. M. Lehtonen O. P. Eerola E. Kero P. (1999). Fecal microflora in healthy infants born by different methods of delivery: permanent changes in intestinal flora after cesarean delivery. J. Pediatr. Gastroenterol. Nutr. 28, 19–25. doi: 10.1097/00005176-199901000-00007, PMID: 9890463

[ref39] Haran J. P. Beaudoin F. L. Suner S. Lu S. (2013). C-reactive protein as predictor of bacterial infection among patients with an influenza-like illness. Am. J. Emerg. Med. 31, 137–144. doi: 10.1016/j.ajem.2012.06.026, PMID: 22944552

[ref40] Hayakawa K. Nishitani K. Tanaka S. (2019). Kynurenine, 3-OH-kynurenine, and anthranilate are nutrient metabolites that alter H3K4 trimethylation and H2AS40 O-GlcNAcylation at hypothalamus-related loci. Sci. Rep. 9:19768. doi: 10.1038/s41598-019-56341-x, PMID: 31875008 PMC6930210

[ref41] Hazra A. Gogtay N. (2017). Biostatistics series module 9: survival analysis. Indian J. Dermatol. 62, 251–257. doi: 10.4103/ijd.IJD_201_17, PMID: 28584366 PMC5448258

[ref42] Heinrich P. C. Castell J. V. Andus T. (1990). Interleukin-6 and the acute phase response. Biochem. J. 265, 621–636. doi: 10.1042/bj2650621, PMID: 1689567 PMC1133681

[ref43] Herberth G. Offenberg K. Rolle-Kampczyk U. Bauer M. Otto W. Röder S.. (2015). Endogenous metabolites and inflammasome activity in early childhood and links to respiratory diseases. J. Allergy Clin. Immunol. 136, 495–497. doi: 10.1016/j.jaci.2015.01.022, PMID: 25754623

[ref44] Hochepied T. Berger F. G. Baumann H. Libert C. (2003). α1-acid glycoprotein: an acute phase protein with inflammatory and immunomodulating properties. Cytokine Growth Factor Rev. 14, 25–34. doi: 10.1016/S1359-6101(02)00054-0, PMID: 12485617

[ref45] Honoré A. H. Aunsbjerg S. D. Ebrahimi P. Thorsen M. Benfeldt C. Knøchel S.. (2016). Metabolic footprinting for investigation of antifungal properties of *Lactobacillus paracasei*. Anal. Bioanal. Chem. 408, 83–96. doi: 10.1007/s00216-015-9103-6, PMID: 26573172

[ref46] Huang W. Cho K. Y. Meng D. Walker W. A. (2021). The impact of indole-3-lactic acid on immature intestinal innate immunity and development: a transcriptomic analysis. Sci. Rep. 11, 1–17. doi: 10.1038/s41598-021-87353-1, PMID: 33850185 PMC8044159

[ref47] Human Metabolome Database (2021). Showing metabocard for L-Tryptophan (HMDB0000929) (2AD). Available at: https://hmdb.ca/metabolites/HMDB0000929 (Accessed August 6, 2021).

[ref48] Illés P. Krasulová K. Vyhlídalová B. Poulíková K. Marcalíková A. Pečinková P.. (2020). Indole microbial intestinal metabolites expand the repertoire of ligands and agonists of the human pregnane X receptor. Toxicol. Lett. 334, 87–93. doi: 10.1016/j.toxlet.2020.09.015, PMID: 33002526

[ref49] Janciauskiene S. M. Bals R. Koczulla R. Vogelmeier C. Köhnlein T. Welte T. (2011). The discovery of α1-antitrypsin and its role in health and disease. Respir. Med. 105, 1129–1139. doi: 10.1016/j.rmed.2011.02.002, PMID: 21367592

[ref50] Ji Y. Gao Y. Chen H. Yin Y. Zhang W. (2019). Indole-3-acetic acid alleviates nonalcoholic fatty liver disease in mice via attenuation of hepatic lipogenesis, and oxidative and inflammatory stress. Nutrients 11:2062. doi: 10.3390/nu11092062, PMID: 31484323 PMC6769627

[ref51] Ji Y. Yin W. Liang Y. Sun L. Yin Y. Zhang W. (2020). Anti-inflammatory and anti-oxidative activity of indole-3-acetic acid involves induction of HO-1 and neutralization of free radicals in RAW264.7 cells. Int. J. Mol. Sci. 21:1579. doi: 10.3390/ijms21051579, PMID: 32106625 PMC7084870

[ref52] Jin U.-H. Cheng Y. Park H. Davidson L. A. Callaway E. S. Chapkin R. S.. (2017). Short chain fatty acids enhance aryl hydrocarbon (Ah) responsiveness in mouse colonocytes and Caco-2 human colon cancer cells. Sci. Rep. 7:10163. doi: 10.1038/s41598-017-10824-x, PMID: 28860561 PMC5579248

[ref53] Kaufmann S. H. E. (2018). Indole propionic acid: a small molecule links between gut microbiota and tuberculosis. Antimicrob. Agents Chemother. 62, e00389–e003818. doi: 10.1128/AAC.00389-18, PMID: 29700005 PMC5923146

[ref54] Kloosterboer S. M. Winter B. C. M. Bahmany S. Al-Hassany L. Dekker A. Dieleman G. C.. (2018). Dried blood spot analysis for therapeutic drug monitoring of antipsychotics: drawbacks of its clinical application. Ther. Drug Monit. 40, 344–350. doi: 10.1097/FTD.0000000000000502, PMID: 29505492

[ref55] Krishnan S. Ding Y. Saedi N. Choi M. Sridharan G. V. Sherr D. H.. (2018). Gut microbiota-derived tryptophan metabolites modulate inflammatory response in hepatocytes and macrophages. Cell Rep. 23, 1099–1111. doi: 10.1016/j.celrep.2018.03.109, PMID: 29694888 PMC6392449

[ref56] Lanis J. M. Alexeev E. E. Curtis V. F. Kitzenberg D. A. Kao D. J. Battista K. D.. (2017). Tryptophan metabolite activation of the aryl hydrocarbon receptor regulates IL-10 receptor expression on intestinal epithelia. Mucosal Immunol. 10, 1133–1144. doi: 10.1038/mi.2016.133, PMID: 28098246 PMC5515702

[ref57] Laurans L. Venteclef N. Haddad Y. Chajadine M. Alzaid F. Metghalchi S.. (2018). Genetic deficiency of indoleamine 2,3-dioxygenase promotes gut microbiota-mediated metabolic health. Nat. Med. 24, 1113–1120. doi: 10.1038/s41591-018-0060-4, PMID: 29942089

[ref58] Le Floc’h N. Otten W. Merlot E. (2011). Tryptophan metabolism, from nutrition to potential therapeutic applications. Amino Acids 41, 1195–1205. doi: 10.1007/s00726-010-0752-7, PMID: 20872026

[ref59] Li H. Illés P. Karunaratne C. V. Nordstrøm L. U. Luo X. Yang A.. (2021). Deciphering structural bases of intestinal and hepatic selectivity in targeting pregnane X receptor with indole-based microbial mimics. Bioorg. Chem. 109:104661. doi: 10.1016/j.bioorg.2021.104661, PMID: 33636438 PMC8646148

[ref60] Li X.-H. Kim S.-K. Lee J.-H. (2017). Anti-biofilm effects of anthranilate on a broad range of bacteria. Sci. Rep. 7:8604. doi: 10.1038/s41598-017-06540-1, PMID: 28819217 PMC5561115

[ref61] Liu Z. Li L. Chen W. Wang Q. Xiao W. Ma Y.. (2018). Aryl hydrocarbon receptor activation maintained the intestinal epithelial barrier function through Notch1 dependent signaling pathway. Int. J. Mol. Med. 41, 1560–1572. doi: 10.3892/ijmm.2017.3341, PMID: 29286081 PMC5819918

[ref62] Lombard G. L. Dowell V. R. (1983). Comparison of three reagents for detecting indole production by anaerobic bacteria in microtest systems. J. Clin. Microbiol. 18, 609–613. doi: 10.1128/jcm.18.3.609-613.1983, PMID: 6630445 PMC270862

[ref63] Mackie R. I. Sghir A. Gaskins H. R. (1999). Developmental microbial ecology of the neonatal gastrointestinal tract. Am. J. Clin. Nutr. 69, 1035S–1045S. doi: 10.1093/ajcn/69.5.1035s, PMID: 10232646

[ref64] Magos L. (1987). Lentner C. (ed.). Geigy scientific tables, 8th Edn. Vol. 1. Units of measurement. Body fluids. Composition of the body. Nutrition. 1981, 298 pp. Vol. 2. Introduction to statistics. Statistical tables. Mathematical formulae. 1982, 241 pp. Vol. 3. Physical chemistry. Composition of the blood. Haematology. Human somatometric data. 1984, 359 pp. Vol. 4. Biochemistry. Metabolism of xenobiotics. Inborn error of metabolism. Pharmacogenetics and ecogenetics. 1986, 330 pp. Ciba-geigy, basel, £12.50 each volume. Distributed in U.K. by Farrand Press. J. Appl. Toxicol. 7:413. doi: 10.1002/jat.2550070617

[ref65] Mändar R. Mikelsaar M. (1996). Transmission of mother’s microflora to the newborn at birth. Biol. Neonate 69, 30–35. doi: 10.1159/000244275, PMID: 8777246

[ref66] Mangge H. Stelzer I. Reininghaus E. Weghuber D. Postolache T. T. Fuchs D. (2014). Disturbed tryptophan metabolism in cardiovascular disease. Curr. Med. Chem. 21, 1931–1937. doi: 10.2174/0929867321666140304105526, PMID: 24606499 PMC4922792

[ref67] Marchini G. Berggren V. Djilali-Merzoug R. Hansson L.-O. (2000). The birth process initiates an acute phase reaction in the fetus-newborn infant. Acta Paediatr. 89, 1082–1086. doi: 10.1111/j.1651-2227.2000.tb03355.x, PMID: 11071089

[ref68] Marcondes A. M. Karoopongse E. Lesnikova M. Margineantu D. Welte T. Dinarello C. A.. (2014). α-1-antitrypsin (AAT)-modified donor cells suppress GVHD but enhance the GVL effect: a role for mitochondrial bioenergetics. Blood 124, 2881–2891. doi: 10.1182/blood-2014-04-570440, PMID: 25224412 PMC4215316

[ref69] Martin R. Nauta A. J. Ben Amor K. Knippels L. M. J. Knol J. Garssen J. (2010). Early life: gut microbiota and immune development in infancy. Benef. Microbes 1, 367–382. doi: 10.3920/BM2010.0027, PMID: 21831776

[ref70] Matsumiya Y. Kato N. Watanabe K. Kato H. (2002). Molecular epidemiological study of vertical transmission of vaginal *Lactobacillus* species from mothers to newborn infants in Japanese, by arbitrarily primed polymerase chain reaction. J. Infect. Chemother. 8, 43–49. doi: 10.1007/s101560200005, PMID: 11957119

[ref71] Mechtler T. P. Stary S. Metz T. F. Jesús V. R. Greber-Platzer S. Pollak A.. (2012). Neonatal screening for lysosomal storage disorders: feasibility and incidence from a nationwide study in Austria. Lancet 379, 335–341. doi: 10.1016/S0140-6736(11)61266-X, PMID: 22133539

[ref72] Meng D. Sommella E. Salviati E. Campiglia P. Ganguli K. Djebali K.. (2020). Indole-3-lactic acid, a metabolite of tryptophan, secreted by *Bifidobacterium longum* subspecies infantis is anti-inflammatory in the immature intestine. Pediatr. Res. 88, 209–217. doi: 10.1038/s41390-019-0740-x, PMID: 31945773 PMC7363505

[ref73] Mimori S. Kawada K. Saito R. Takahashi M. Mizoi K. Okuma Y.. (2019). Indole-3-propionic acid has chemical chaperone activity and suppresses endoplasmic reticulum stress-induced neuronal cell death. Biochem. Biophys. Res. Commun. 517, 623–628. doi: 10.1016/j.bbrc.2019.07.074, PMID: 31378367

[ref74] Mitsou E. K. Kirtzalidou E. Oikonomou I. Liosis G. Kyriacou A. (2008). Fecal microflora of Greek healthy neonates. Anaerobe 14, 94–101. doi: 10.1016/j.anaerobe.2007.11.002, PMID: 18207437

[ref75] Natividad J. M. Agus A. Planchais J. Lamas B. Jarry A. C. Martin R.. (2018). Impaired aryl hydrocarbon receptor ligand production by the gut microbiota is a key factor in metabolic syndrome. Cell Metab. 28, 737–749. doi: 10.1016/j.cmet.2018.07.001, PMID: 30057068

[ref76] Neu J. Rushing J. (2011). Cesarean versus vaginal delivery: long-term infant outcomes and the hygiene hypothesis. Clin. Perinatol. 38, 321–331. doi: 10.1016/j.clp.2011.03.008, PMID: 21645799 PMC3110651

[ref77] Nikolaus S. Schulte B. Al-Massad N. Thieme F. Schulte D. M. Bethge J.. (2017). Increased tryptophan metabolism is associated with activity of inflammatory bowel diseases. Gastroenterology 153, 1504.e2–1516.e2. doi: 10.1053/j.gastro.2017.08.028, PMID: 28827067

[ref78] Okamura M. Shizu R. Abe T. Kodama S. Hosaka T. Sasaki T.. (2020). PXR functionally interacts with NF-κB and AP-1 to downregulate the inflammation-induced expression of chemokine CXCL2 in mice. Cell 9:2296. doi: 10.3390/cells9102296, PMID: 33076328 PMC7602528

[ref79] Pajor A. M. Sun N. N. (2013). Nonsteroidal anti-inflammatory drugs and other anthranilic acids inhibit the Na+/dicarboxylate symporter from *Staphylococcus aureus*. Biochemistry 52, 2924–2932. doi: 10.1021/bi301611u, PMID: 23566164

[ref80] Palmer C. Bik E. M. DiGiulio D. B. Relman D. A. Brown P. O. (2007). Development of the human infant intestinal microbiota. PLoS Biol. 5:e177. doi: 10.1371/journal.pbio.0050177, PMID: 17594176 PMC1896187

[ref81] Pavlova T. Vidova V. Bienertova-Vasku J. Janku P. Almasi M. Klanova J.. (2017). Urinary intermediates of tryptophan as indicators of the gut microbial metabolism. Anal. Chim. Acta 987, 72–80. doi: 10.1016/j.aca.2017.08.022, PMID: 28916042

[ref82] Penders J. Thijs C. Vink C. Stelma F. F. Snijders B. Kummeling I.. (2006). Factors influencing the composition of the intestinal microbiota in early infancy. Pediatrics 118, 511–521. doi: 10.1542/peds.2005-2824, PMID: 16882802

[ref83] Prendergast G. C. Chang M. Y. Mandik-Nayak L. Metz R. Muller A. J. (2011). Indoleamine 2,3-dioxygenase as a modifier of pathogenic inflammation in cancer and other inflammation-associated diseases. Curr. Med. Chem. 18, 2257–2262. doi: 10.2174/092986711795656072, PMID: 21517753 PMC4384691

[ref84] Roager H. M. Licht T. R. (2018). Microbial tryptophan catabolites in health and disease. Nat. Commun. 9:3294. doi: 10.1038/s41467-018-05470-4, PMID: 30120222 PMC6098093

[ref85] Romagnani S. (2006). Regulation of the T cell response. Clin. Exp. Allergy 36, 1357–1366. doi: 10.1111/j.1365-2222.2006.02606.x, PMID: 17083345

[ref86] Roth W. Zadeh K. Vekariya R. Ge Y. Mohamadzadeh M. (2021). Tryptophan metabolism and gut-brain homeostasis. Int. J. Mol. Sci. 22, 1–23. doi: 10.3390/ijms22062973, PMID: 33804088 PMC8000752

[ref87] Russell W. R. Duncan S. H. Scobbie L. Duncan G. Cantlay L. Calder A. G.. (2013). Major phenylpropanoid-derived metabolites in the human gut can arise from microbial fermentation of protein. Mol. Nutr. Food Res. 57, 523–535. doi: 10.1002/mnfr.201200594, PMID: 23349065

[ref88] Sain-van der Velden M. G. M. van der Ham M. Gerrits J. Prinsen H. C. M. T. Willemsen M. Pras-Raves M. L.. (2017). Quantification of metabolites in dried blood spots by direct infusion high resolution mass spectrometry. Anal. Chim. Acta 979, 45–50. doi: 10.1016/j.aca.2017.04.038, PMID: 28599708

[ref89] Sanidad K. Z. Zeng M. Y. (2020). Neonatal gut microbiome and immunity. Curr. Opin. Microbiol. 56, 30–37. doi: 10.1016/j.mib.2020.05.011, PMID: 32634598 PMC8729197

[ref90] Scott S. A. Fu J. Chang P. V. (2020). Microbial tryptophan metabolites regulate gut barrier function via the aryl hydrocarbon receptor. Proc. Natl. Acad. Sci. U. S. A. 117, 19376–19387. doi: 10.1073/pnas.2000047117, PMID: 32719140 PMC7431026

[ref91] Shao Y. Forster S. C. Tsaliki E. Vervier K. Strang A. Simpson N.. (2019). Stunted microbiota and opportunistic pathogen colonization in caesarean-section birth. Nature 574, 117–121. doi: 10.1038/s41586-019-1560-1, PMID: 31534227 PMC6894937

[ref92] Sirianni A. C. Jiang J. Zeng J. Mao L. L. Zhou S. Sugarbaker P.. (2015). N-acetyl-l-tryptophan, but not N-acetyl-d-tryptophan, rescues neuronal cell death in models of amyotrophic lateral sclerosis. J. Neurochem. 134, 956–968. doi: 10.1111/jnc.13190, PMID: 26031348

[ref93] Smith E. A. Macfarlane G. T. (1996). Enumeration of human colonie bacteria producing phenolic and indolic compounds: effects of pH, carbohydrate availability and retention time on dissimilatory aromatic amino acid metabolism. J. Appl. Bacteriol. 81, 288–302. doi: 10.1111/j.1365-2672.1996.tb04331.x, PMID: 8810056

[ref94] Tests for Normality [R package nortest version 1.0-4] (2021). Comprehensive R Archive Network (CRAN)UR. Available at: https://cran.r-project.org/web/packages/nortest/index.html (Accessed August 6, 2021).

[ref95] Tonelli D. Gattavecchia E. Gandolfi M. (1982). Thin-layer chromatographic determination of indolic tryptophan metabolites in human urine using Sep-Pak C18 extraction. J. Chromatogr. 231, 283–289. doi: 10.1016/s0378-4347(00)81853-8, PMID: 7130309

[ref96] Torun M. M. Bahar H. Gür E. Taştan Y. Alikaşifoğlu M. Arvas A. (2002). Anaerobic fecal flora in healthy breast-fed Turkish babies born by different methods. Anaerobe 8, 63–67. doi: 10.1006/anae.2002.0415

[ref97] Various R Programming Tools for Plotting Data [R package gplots version 3.0.4] (2021). Comprehensive R Archive Network (CRAN)UR. Available at: https://cran.r-project.org/web/packages/gplots/index.html (Accessed August 6, 2021).

[ref98] Venkatesh M. Mukherjee S. Wang H. Li H. Sun K. Benechet A. P.. (2014). Symbiotic bacterial metabolites regulate gastrointestinal barrier function via the xenobiotic sensor PXR and toll-like receptor 4. Immunity 41, 296–310. doi: 10.1016/j.immuni.2014.06.014, PMID: 25065623 PMC4142105

[ref99] Vidova V. Spacil Z. (2017). A review on mass spectrometry-based quantitative proteomics: targeted and data independent acquisition. Anal. Chim. Acta 964, 7–23. doi: 10.1016/j.aca.2017.01.059, PMID: 28351641

[ref100] Vidova V. Stuchlikova E. Vrbova M. Almasi M. Klanova J. Thon V.. (2019). Multiplex assay for quantification of acute phase proteins and immunoglobulin A in dried blood spots. J. Proteome Res. 18, 380–391. doi: 10.1021/acs.jproteome.8b00657, PMID: 30408962

[ref101] Visualization of a Correlation Matrix [R package corrplot version 0.84] (2021). Comprehensive R Archive Network (CRAN)UR. Available at: https://cran.r-project.org/web/packages/corrplot/index.html (Accessed August 6, 2021).

[ref102] Wall R. Ross R. P. Ryan C. A. Hussey S. Murphy B. Fitzgerald G. F.. (2009). Role of gut microbiota in early infant development. Clin. Med. Pediatr. 3, 45–54. doi: 10.4137/cmped.s2008, PMID: 23818794 PMC3676293

[ref103] Wampach L. Heintz-Buschart A. Fritz J. V. Ramiro-Garcia J. Habier J. Herold M.. (2018). Birth mode is associated with earliest strain-conferred gut microbiome functions and immunostimulatory potential. Nat. Commun. 9:5091. doi: 10.1038/s41467-018-07631-x, PMID: 30504906 PMC6269548

[ref104] Wickham H. (2009). ggplot2. New York, NY: Springer-Verlag.

[ref105] Wikoff W. R. Anfora A. T. Liu J. Schultz P. G. Lesley S. A. Peters E. C.. (2009). Metabolomics analysis reveals large effects of gut microflora on mammalian blood metabolites. Proc. Natl. Acad. Sci. U. S. A. 106, 3698–3703. doi: 10.1073/pnas.0812874106, PMID: 19234110 PMC2656143

[ref106] Williams B. B. van Benschoten A. H. Cimermancic P. Donia M. S. Zimmermann M. Taketani M.. (2014). Discovery and characterization of gut microbiota decarboxylases that can produce the neurotransmitter tryptamine. Cell Host Microbe 16, 495–503. doi: 10.1016/j.chom.2014.09.001, PMID: 25263219 PMC4260654

[ref107] Wlodarska M. Luo C. Kolde R. d’Hennezel E. Annand J. W. Heim C. E.. (2017). Indoleacrylic acid produced by commensal peptostreptococcus species suppresses inflammation. Cell Host Microbe 22, 25.e6–37.e6. doi: 10.1016/j.chom.2017.06.007, PMID: 28704649 PMC5672633

[ref108] Wyatt M. Greathouse K. L. (2021). Targeting dietary and microbial tryptophan-indole metabolism as therapeutic approaches to colon cancer. Nutrients 13:1189. doi: 10.3390/nu13041189, PMID: 33916690 PMC8066279

[ref109] Yamada T. Wada A. Yamaguchi T. Itoh Y. Kawai T. (1997). Automated measurement of a constitutive isotype of serum amyloid A/SAA4 and comparison with other apolipoproteins. J. Clin. Lab. Anal. 11, 363–368. doi: 10.1002/(SICI)1098-2825(1997)11:6<363::AID-JCLA10>3.0.CO;2-U, PMID: 9406058 PMC6760705

[ref110] Zachary J. F. (2017). Pathologic Basis of Veterinary Disease. 6th Edn. eds. Zachary J. F. McGavin M. D. (Mosby).

[ref111] Zelante T. Iannitti R. G. Cunha C. Luca A. Giovannini G. Pieraccini G.. (2013). Tryptophan catabolites from microbiota engage aryl hydrocarbon receptor and balance mucosal reactivity via interleukin-22. Immunity 39, 372–385. doi: 10.1016/j.immuni.2013.08.003, PMID: 23973224

[ref112] Zhang Z. Tang H. Chen P. Xie H. Tao Y. (2019). Demystifying the manipulation of host immunity, metabolism, and extraintestinal tumors by the gut microbiome. Signal Transduct. Target. Ther. 4:41. doi: 10.1038/s41392-019-0074-5, PMID: 31637019 PMC6799818

[ref113] Zhao S. Guo Y. Sheng Q. Shyr Y. (2014). Heatmap3: an improved heatmap package with more powerful and convenient features. BMC Bioinformatics 15(Suppl. 10):P16. doi: 10.1186/1471-2105-15-S10-P16

[ref114] Zhou C. Tabb M. Nelson E. Grün F. Verma S. Sandatrafiei A.. (2006). Mutual repression between steroid and xenobiotic receptor and NF-kappaB signaling pathways links xenobiotic metabolism and inflammation. J. Clin. Invest. 116, 2280–2289. doi: 10.1172/JCI26283, PMID: 16841097 PMC1501109

[ref115] Zihni C. Mills C. Matter K. Balda M. S. (2016). Tight junctions: from simple barriers to multifunctional molecular gates. Nat. Rev. Mol. Cell Biol. 17, 564–580. doi: 10.1038/nrm.2016.80, PMID: 27353478

