# Supplementary material for: Profiling Tryptophan Catabolites of Human Gut Microbiota and Acute-Phase Protein Levels in Neonatal Dried Blood Specimens
Source: Front Microbiol. 2021 Oct 27;12:665743. doi: 10.3389/fmicb.2021.665743 (PMC8581761; doi:10.3389/fmicb.2021.665743)

Supplementary Material

Profiling Tryptophan Catabolites of Human Gut Microbiota and Acute-Phase Protein Levels in Neonatal Dried Blood Specimens

Anne-Christine Aust^1#^, Eliska Benesova^1#^, Veronika Vidova^1^, Katerina Coufalikova^1^, Sona Smetanova^1^, Ivo Borek^2^, Petr Janku^3^, Eva Budinska^1^, Jana Klanova^1^, Vojtech Thon^1^, Zdenek Spacil*^1^

^1^RECETOX, Faculty of Science, Masaryk University, Brno, Czech Republic

^2^ Department of Pediatrics, University Hospital Brno and Masaryk University Medical School, Brno, Czech Republic

^3^ Department of Gynecology and Obstetrics, University Hospital Brno and Masaryk University Medical School, Brno, Czech Republic

# Authors contributed equally.

* Corresponding author

Address correspondence to Dr. Zdenek Spacil, Masaryk University, Faculty of Science, Kamenice 753/5, pavilion D29/418, 625 00 Brno, Czech Republic. Tel. (+420) 549 49 7989; e-mail: [spacil@recetox.muni.cz](mailto:spacil@recetox.muni.cz) or [spacil@u.washington.edu](mailto:spacil@u.washington.edu)

# Table of Contents

Materials and Methods - Mass Spectrometry Data Processing.

Materials and Methods – Linearity.

Table S-1: Characteristics of individual neonates participating in the study.

Table S-2: Average, minimal and maximal values of the neonatal birth weight, birth length, gestational age, Apgar score, and dried blood specimen sampling time.

Table S-3: Synthetic peptide internal standards for protein quantification.

Table S-4: Metabolite internal standards for selected reaction monitoring assay, quantifier transitions, and validation parameters.

Table S-5: Selected reaction monitoring assay library for metabolites.

Table S-6: Linear response concentration range for metabolite internal standards.

Figure S-1: Flowchart of dried blood specimen processing protocol.

Figure S-2: Typical chromatograms of tryptophan catabolites in dried blood specimens.

Figure S-3: Matrix-matched calibration curves for metabolite internal standards.

Figure S-4: Heatmap with all metabolites and proteins quantified in dried blood specimens.

Figure S-5: Heatmap with all proteins quantified in dried blood specimens.

Figure S-6: SAA1/2, SAA4, and A1AG1 blood concentrations are grouped relative to the limit of detection (LOD) for CRP (a) or SAA1 (b), p<0.0001.

Figure S-7: Metabolites and proteins correlation matrix plots for vaginally (VD) and caesarian (CD) born neonates.

Figure S-8: ILA and IAA correlation within all dried blood specimens and grouped by neonates' mode of delivery (VD for vaginal and CD for Cesarean delivery).

Mass Spectrometry Data Processing. The area under a chromatographic peak (AUC) was used to calculate concentration (nM and mg/L). The AUC and the known concentration of labeled internal standards were used to calculate the LOD (Equation 2) and LOQ (Equation 3) using the slope of the calibration curve.

$$LOD=\frac{SD*3}{slope}$$

*Equation 2: Calculation for the Limit of Detection for labeled standard.*

$$LOQ=\frac{SD*10}{slope}$$

*Equation 3: Calculation of the Limit of Quantification for labeled standards.*

For non-labeled standards, the response factor determined for each analyte was used to calculate respective LOD (Equation 4) and LOQ (Equation 5).

$$LOD=\frac{{LOD}_{labelled metabolite}}{{RF}_{labelled metabolite}}$$

*Equation 4: Calculation of Limit of Detection for non-labeled metabolites.*

$$LOQ=\frac{{LOQ}_{labelled metabolite}}{{RF}_{labelled metabolite}}$$

*Equation 5: Calculation of Limit of Quantification for non-labeled metabolites.*

Linearity. The measured metabolites were normalized through the ratio between an unlabeled and a labeled metabolite peak area. The normalized metabolite area was plotted against the metabolite concentration of unlabeled metabolites. The area measured for each dilution was plotted against the concentration of the non-labeled metabolite. For every labeled standard in the linearity curve, the Coefficient of variation (CV) was calculated (Equation 6).

Coefficient of Variation =$\frac{\bar{x}_{Area of sample} x 100}{s_{Standard deviation of each dilution step}}$

*Equation 6: Calculation of Coefficient of Variation (CV)*

**Table S-1: Characteristics of individual neonates participating in the study.**

| **Sample** | **Gestation age** | **Mode of delivery** | **Medication** | **Anamnesis of mother** | **Anamnesis of neonate** | **Sex** | **Length (cm)** | **Weight (g)** | **Apgar score** |
| --- | --- | --- | --- | --- | --- | --- | --- | --- | --- |
| 1 | 40+2 | vaginal | --- | --- | --- | male | 51 | 3600 | [9, 10, 10] |
| 2 | 39+6 | vaginal | epidural | --- | erythema | male | 50 | 3240 | [10, 10, 10] |
| 3 | 40+4 | vaginal | --- | --- | intrauterine hypoxia, traumatism | female | 52 | 3410 | [6, 9, 10] |
| 4 | 40+4 | vaginal | --- | anemia | erythema | male | 49 | 3240 | [9, 9, 10] |
| 5 | 41+0 | vaginal | --- | --- | jaundice, traumatism | female | 50 | 3760 | [8, 9, 9] |
| 6 | 39+6 | C. section | --- | --- | erythema | female | 47 | 3220 | [10, 10, 10] |
| 7 | 39+3 | vaginal | oxytocin | anemia | intrauterine hypoxia | male | 51 | 3230 | [3, 7, 9] |
| 8 | 40+1 | vaginal | --- | --- | --- | male | 52 | 3630 | [10, 10, 10] |
| 9 | 40+1 | vaginal | --- | anemia, diabetes | --- | female | 49 | 3320 | [10, 10, 10] |
| 10 | 39+1 | vaginal | --- | asthma | --- | male | 50 | 3240 | [10, 10, 10] |
| 11 | 40+0 | vaginal | --- | anemia | erythema | female | 49 | 3820 | [10, 10, 10] |
| 12 | 41+3 | C. section | --- | --- | erythema | male | 49 | 3250 | [9, 9, 10] |
| 13 | 39+3 | C. section | --- | --- | jaundice | male | 46 | 2490 | [5, 8, 8] |
| 14 | 40+2 | vaginal | oxytocin | --- | --- | female | 49 | 3320 | [10, 10, 10] |
| 15 | 41+0 | vaginal | oxytocin | streptococcus | --- | female | 49 | 3690 | [9, 10, 10] |
| 16 | 40+2 | vaginal | epidural | --- | intrauterine hypoxia | male | 50 | 3360 | [10, 10, 10] |
| 17 | 40+5 | C. section | epidural | asthma | --- | male | 51 | 3900 | [7, 7, 8] |
| 18 | 40+4 | vaginal | --- | --- | --- | male | 54 | 3910 | [10, 10, 10] |
| 19 | 40+5 | vaginal | --- | anemia, streptococcus | --- | female | 50 | 3600 | [9, 10, 10] |
| 20 | 39+5 | C. section | --- | --- | --- | female | 51 | 3790 | [9, 10, 10] |
| 21 | 40+0 | vaginal | --- | --- | intrauterine hypoxia | male | 52 | 3800 | [10, 10, 10] |
| 22 | 38+5 | vaginal | --- | --- | intrauterine hypoxia, erythema | female | 48 | 2750 | [9, 9, 9] |
| 23 | 39+3 | vaginal | --- | --- | --- | male | 52 | 3950 | [9, 9, 10] |
| 24 | 40+0 | vaginal | --- | --- | --- | male | 48 | 3200 | [9, 10, 10] |
| 25 | 39+3 | vaginal | epidural | anemia | jaundice | female | 52 | 3520 | [9, 10, 10] |
| 26 | 38+6 | vaginal | --- | --- | erythema, traumatism | male | 49 | 3030 | [9, 10, 10] |
| 27 | 39+5 | vaginal | --- | --- | --- | male | 53 | 4100 | [10, 10, 10] |
| 28 | 39+4 | vaginal | --- | --- | jaundice | female | 48 | 3040 | [10, 10, 10] |
| 29 | 40+4 | vaginal | epidural | --- | intrauterine hypoxia, jaundice | male | 51 | 3730 | [9, 10, 10] |
| 30 | 40+3 | vaginal | entonox | anemia | --- | male | 60 | 3390 | [9, 10, 10] |
| 31 | 40+3 | vaginal | --- | anemia | erythema, jaundice | male | 51 | 3770 | [9, 10, 10] |
| 32 | 39+2 | vaginal | epidural | asthma | erythema, traumatism | male | 48 | 2960 | [10, 10, 10] |
| 33 | 39+4 | vaginal | epidural | --- | --- | female | 49 | 3210 | [10, 10, 10] |
| 34 | 40+2 | vaginal | --- | --- | --- | male | 49 | 3370 | [9, 10, 10] |
| 35 | 40+5 | vaginal | oxytocin | --- | --- | female | 49 | 3200 | [9, 10, 10] |
| 36 | 39+4 | vaginal | --- | --- | intrauterine hypoxia | male | 50 | 3360 | [9, 10, 10] |
| 37 | 39+5 | vaginal | epidural | anemia, streptococcus | intrauterine hypoxia | female | 50 | 3650 | [8, 10, 10] |
| 38 | 38+6 | vaginal | --- | anemia | --- | male | 49 | 3260 | [9, 10, 10] |
| 39 | 40+2 | vaginal | --- | --- | --- | male | 52 | 3540 | [10, 10, 10] |
| 40 | 39+6 | vaginal | epidural | --- | jaundice | female | 51 | 3550 | [9, 9, 9] |
| 41 | 40+2 | vaginal | --- | anemia | congenital pneumonia | male | 51 | 3590 | [8, 9, 9] |
| 42 | 40+2 | vaginal | epidural | anemia | --- | female | 50 | 3700 | [9, 9, 10] |
| 43 | 39+5 | vaginal | --- | --- | jaundice | male | 51 | 2900 | [9, 10, 10] |
| 44 | 39+5 | vaginal | epidural | asthma | erythema, jaundice | male | 53 | 3540 | [8, 10, 10] |
| 45 | 39+3 | C. section | --- | streptococcus | --- | female | 50 | 2880 | [10, 10, 10] |
| 46 | 39+1 | vaginal | --- | asthma | jaundice | male | 51 | 3740 | [9, 10, 10] |
| 47 | 40+6 | vaginal | --- | --- | erythema | male | 51 | 4180 | [5, 8, 9] |
| 48 | 39+0 | C. section | oxytocin | anemia | --- | female | 50 | 3000 | [10, 10, 10] |
| 49 | 40+4 | vaginal | epidural | --- | --- | female | 51 | 3460 | [10, 10, 10] |
| 50 | 40+1 | vaginal | --- | --- | traumatism | female | 50 | 3120 | [10, 10, 10] |
| 51 | 40+0 | C. section | --- | --- | --- | male | 49 | 3780 | [9, 10, 10] |
| 52 | 39+3 | vaginal | epidural | asthma | erythema, traumatism | male | 51 | 2870 | [9, 10, 10] |
| 53 | 40+3 | vaginal | --- | anemia | erythema | male | 52 | 3550 | [10, 10, 10] |
| 54 | 40+2 | vaginal | --- | --- | traumatism | female | 47 | 2810 | [9, 10, 10] |
| 55 | 40+2 | vaginal | --- | anemia | --- | female | 51 | 3380 | [10, 10, 10] |
| 56 | 39+6 | vaginal | epidural | --- | intrauterine hypoxia, erythema | female | 52 | 3340 | [9, 10, 10] |
| 57 | 40+4 | vaginal | epidural | --- | --- | male | 55 | 4190 | [9, 10, 10] |
| 58 | 40+5 | vaginal | epidural | --- | --- | female | 49 | 2670 | [10, 10, 10] |
| 59 | 40+1 | vaginal | --- | --- | --- | male | 52 | 3420 | [9, 10, 10] |
| 60 | 41+1 | vaginal | --- | --- | erythema | female | 55 | 4290 | [9, 9, 10] |
| 61 | 40+6 | vaginal | epidural | streptococcus | intrauterine hypoxia | female | 50 | 3220 | [9, 9, 10] |
| 62 | 40+3 | C. section | oxytocin | --- | intrauterine hypoxia | male | 53 | 4900 | [10, 10, 10] |
| 63 | 40+5 | vaginal | --- | --- | --- | male | 49 | 3300 | [10, 10, 10] |
| 64 | 38+5 | vaginal | --- | anemia | jaundice | female | 49 | 2750 | [9, 9, 10] |
| 65 | 40+6 | vaginal | --- | --- | --- | female | 52 | 3860 | [9, 9, 9] |
| 66 | 40+3 | vaginal | epidural | --- | intrauterine hypoxia, erythema | male | 54 | 3740 | [7, 9, 9] |
| 67 | 40+3 | vaginal | entonox | streptococcus | --- | male | 50 | 3030 | [10, 10, 10] |
| 68 | 39+4 | vaginal | --- | --- | erythema | male | 50 | 3380 | [9, 9, 9] |
| 69 | 38+5 | C. section | epidural | --- | intrauterine hypoxia, traumatism | male | 49 | 4070 | [9, 9, 9] |
| 70 | 40+5 | vaginal | epidural | --- | intrauterine hypoxia | female | 51 | 3300 | [9, 10, 10] |
| 71 | 40+1 | vaginal | epidural | --- | intrauterine hypoxia, traumatism | male | 49 | 3280 | [9, 9, 9] |
| 72 | 40+0 | vaginal | nalbuphine | --- | intrauterine hypoxia, erythema | female | 48 | 2840 | [9, 9, 10] |
| 73 | 40+6 | vaginal | --- | --- | --- | male | 51 | 3570 | [8, 9, 10] |
| 74 | 39+2 | vaginal | --- | --- | --- | male | 50 | 3150 | [10, 10, 10] |
| 75 | 41+2 | vaginal | --- | --- | erythema, jaundice | male | 51 | 3580 | [5, 8, 9] |
| 76 | 40+5 | vaginal | --- | --- | jaundice | female | 50 | 3120 | [5, 7, 9] |
| 77 | 40+2 | vaginal | oxytocin | streptococcus | erythema | male | 52 | 4350 | [8, 9, 10] |
| 78 | 41+0 | vaginal | entonox | --- | --- | male | 50 | 3420 | [9, 10, 10] |
| 79 | 40+1 | vaginal | oxytocin | streptococcus | intrauterine hypoxia | male | 52 | 3290 | [5, 7, 8] |
| 80 | 40+5 | C. section | epidural | streptococcus | intrauterine hypoxia | male | 52 | 3700 | [9, 10, 10] |
| 81 | 39+4 | vaginal | --- | anemia | intrauterine hypoxia | female | 50 | 4140 | [9, 10, 10] |
| 82 | 40+5 | vaginal | epidural | streptococcus | --- | female | 52 | 3440 | [8, 10, 10] |
| 83 | 41+1 | vaginal | epidural | anemia | traumatism | female | 52 | 3930 | [9, 9, 10] |
| 84 | 40+5 | vaginal | epidural | --- | erythema | male | 51 | 3790 | [10, 10, 10] |
| 85 | 40+3 | vaginal | --- | anemia | erythema | female | 52 | 3760 | [10, 10, 10] |
| 86 | 40+4 | vaginal | --- | --- | erythema | female | 52 | 3640 | [10, 10, 10] |
| 87 | 40+0 | vaginal | --- | --- | erythema | male | 50 | 2850 | [9, 9, 10] |
| 88 | 39+2 | C. section | --- | --- | intrauterine hypoxia | male | 50 | 3810 | [1, 6, 8] |
| 89 | 38+6 | vaginal | --- | --- | --- | male | 50 | 3530 | [9, 10, 10] |
| 90 | 38+6 | vaginal | --- | anemia | erythema | female | 50 | 3430 | [10, 10, 10] |
| 91 | 40+0 | vaginal | --- | streptococcus | --- | female | 47 | 3230 | [10, 10, 10] |
| 92 | 40+4 | vaginal | epidural | --- | erythema | male | 52 | 3810 | [9, 10, 10] |
| 93 | 39+2 | vaginal | --- | --- | --- | female | 50 | 3250 | [10, 10, 10] |
| 94 | 39+2 | vaginal | --- | --- | --- | male | 50 | 3900 | [9, 10, 10] |
| 95 | 39+2 | C. section | --- | --- | --- | male | 49 | 3130 | [9, 9, 10] |
| 96 | 41+3 | vaginal | --- | --- | --- | female | 50 | 3700 | [9, 10, 10] |
| 97 | 38+6 | C. section | remifentanil | --- | erythema, jaundice | female | 50 | 3950 | [10, 10, 10] |
| 98 | 39+3 | vaginal | oxytocin | --- | --- | female | 51 | 3510 | [10, 10, 10] |
| 99 | 40+5 | vaginal | epidural | anemia, diabetes | intrauterine hypoxia | male | 50 | 3750 | [9, 9, 9] |
| 100 | 39+6 | vaginal | oxytocin | --- | jaundice | male | 50 | 3200 | [9, 10, 10] |
| 101 | 39+3 | vaginal | --- | --- | --- | female | 54 | 3820 | [9, 10, 10] |
| 102 | 40+1 | vaginal | --- | diabetes | intrauterine hypoxia, jaundice | female | 51 | 4010 | [9, 10, 10] |
| 103 | 41+0 | vaginal | --- | streptococcus | erythema | male | 51 | 3730 | [9, 10, 10] |
| 104 | 40+2 | vaginal | epidural | --- | erythema | male | 50 | 3300 | [8, 9, 10] |
| 105 | 40+1 | vaginal | --- | --- | jaundice | male | 51 | 3590 | [10, 10, 10] |
| 106 | 39+1 | C. section | --- | anemia | intrauterine hypoxia | male | 47 | 2960 | [1, 0, 0] |
| 107 | 40+2 | vaginal | epidural | --- | --- | male | 50 | 3000 | [10, 10, 10] |
| 108 | 40+0 | C. section | epidural | --- | erythema | female | 47 | 2930 | [9, 10, 10] |
| 109 | 39+4 | C. section | --- | --- | --- | male | 47 | 3300 | [9, 10, 10] |
| 110 | 39+0 | vaginal | --- | anemia | erythema | male | 47 | 2470 | [9, 10, 10] |
| 111 | 41+3 | vaginal | epidural | --- | --- | male | 51 | 4190 | [10, 10, 10] |
| 112 | 41+0 | vaginal | --- | asthma | --- | female | 53 | 3550 | [8, 8, 9] |
| 113 | 39+5 | vaginal | oxytocin | anemia, asthma, diabetes | --- | female | 50 | 3730 | [9, 10, 10] |
| 114 | 39+3 | C. section | --- | --- | --- | male | 50 | 2960 | [9, 9, 9] |
| 115 | 41+1 | vaginal | --- | anemia | intrauterine hypoxia | male | 52 | 3170 | [8, 10, 10] |
| 116 | 40+3 | vaginal | --- | diabetes | --- | female | 51 | 3680 | [9, 10, 10] |
| 117 | 40+4 | vaginal | --- | --- | --- | male | 50 | 4130 | [10, 10, 10] |
| 118 | 40+4 | vaginal | --- | anemia | erythema | female | 51 | 4110 | [9, 9, 10] |
| 119 | 39+2 | vaginal | --- | --- | --- | male | 48 | 3180 | [9, 10, 10] |
| 120 | 40+0 | vaginal | --- | streptococcus | --- | female | 51 | 3820 | [9, 9, 9] |
| 121 | 40+6 | vaginal | --- | --- | --- | female | 53 | 4450 | [9, 10, 10] |
| 122 | 39+0 | vaginal | --- | --- | --- | male | 48 | 3230 | [9, 9, 9] |
| 123 | 38+2 | C. section | epidural | anemia | --- | male | 49 | 3230 | [9, 10, 10] |
| 124 | 41+1 | C. section | --- | --- | --- | female | 53 | 3930 | [10, 10, 10] |
| 125 | 40+2 | vaginal | --- | --- | --- | male | 51 | 4000 | [10, 10, 10] |
| 126 | 40+6 | vaginal | --- | --- | erythema, jaundice | male | 53 | 4130 | [7, 9, 10] |
| 127 | 40+1 | vaginal | --- | --- | --- | female | 52 | 3620 | [9, 9, 10] |
| 128 | 39+4 | vaginal | --- | --- | jaundice | male | 49 | 3450 | [7, 8, 9] |
| 129 | 40+6 | vaginal | epidural | --- | jaundice, traumatism | female | 52 | 3690 | [10, 10, 10] |
| 130 | 40+2 | vaginal | entonox | --- | --- | male | 50 | 3380 | [9, 9, 9] |
| 131 | 40+4 | vaginal | oxytocin | --- | intrauterine hypoxia | male | 54 | 4560 | [9, 10, 10] |
| 132 | 39+6 | vaginal | oxytocin | anemia | erythema, jaundice, traumatism | male | 50 | 3090 | [9, 10, 10] |
| 133 | 39+5 | vaginal | --- | --- | erythema | female | 49 | 2980 | [9, 10, 10] |
| 134 | 40+1 | vaginal | --- | diabetes | intrauterine hypoxia, jaundice | male | 52 | 3430 | [10, 10, 10] |

**Table S-2: Average, minimal and maximal values of the neonatal birth weight, birth length, gestational age, Apgar score, and dried blood specimen sampling time.**

|  | **VD** | | | **CD** | | |
| --- | --- | --- | --- | --- | --- | --- |
|  | **Average ± SD** | **minimal value** | **maximal value** | **Average ± SD** | **minimal value** | **maximal value** |
| **Birth length (cm)** | 51 ± 2 | 47 | 60 | 49 ± 2 | 46 | 53 |
| **Birth weight (g)** | 3500 ± 401 | 2470 | 4560 | 3459 ± 564 | 2490 | 4900 |
| **Gestation age (weeks+days)** | [40+1] ± [0+4] | [38+5] | [41+3] | [39+5] ± [0+6] | [38+2] | [41+2] |
| **Apgar score** | [9,10,10] ± [1,1,0] | [3,7,8] | [10,10,10] | [8,9,9] ± [3,2,2] | [1,0,0]* | [10,10,10] |
| **DBS sampling (hours after delivery)** | 62 ± 10 | 44 | 126 | 66 ± 5 | 53 | 72 |

* Neonate born via acute C. section

# Table S-3. Synthetic peptide internal standards for protein quantification. All peptides are labeled on C-terminal arginine (R*. ^13^C_6_H_14_O_2_^15^N_4_) or lysine (K*. ^13^C_6_H_14_O_2_^15^N_2_) and equipped with a tetrapeptide (serine-alanine-nitrotyrosine-glycine) trypsin cleavable tag.

| **Protein** | **Sequence** | **MW** | **Spiked concentration**  **[nM]** | **Quantifier SRM transition** | **Retention time (min)** | **Calibration** | | |
| --- | --- | --- | --- | --- | --- | --- | --- | --- |
|  |  |  |  |  |  | **LOD (mg/L)** | **LOQ (mg/L)** | **R^2^** |
| SAA1 | FFGHGAEDSLADQAANEWG**R**- SAnYG | 2611.6 | 100 | 730.0 -> 742.4 | 13.4 | 1.4 | 4.4 | 0.9973 |
| SAA2-1 | GAEDSLADQAAN**K**-SAnYG | 1720.6 | 100 | 649.3 -> 725.4 | 4.0 | 0.5 | 1.7 | 0.9990 |
|  | LTGHGAEDSLADQAAN**K**-SAnYG | 2129.1 | 100 | 569.3 -> 725.4 | 3.8 | 0.4 | 1.4 | 0.9991 |
|  | GPGGAWAAEVISNA**R-**SAnYG | 1888.9 | 250 | 733.4 -> 940.5 | 13.4 | 19.1 | 57.9 | 0.9936 |
| SAA1/2 | SFFSFLGEAFDGA**R**-SAnYG | 1984.0 | 400 | 780.9 -> 832.4 | 24.1 | 2.9 | 8.8 | 0.9906 |
| SAA4 | EALQGVGDMG**R**-SAnYG | 1565.5 | 100 | 571.8 -> 701.3 | 6.6 | 2.9 | 8.6 | 0.9997 |
|  | FRPDGLP**K**-SAnYG | 1360.4 | 100 | 313.2 -> 317.7 | 3.9 | 0.3 | 0.9 | 0.9939 |
| CRP | ESDTSYVSL**K**-SAnYG | 1559.5 | 50 | 568.8 -> 704.4 | 6.4 | 2.2 | 6.8 | 0.9930 |
| A1AT-1 | AVLTIDE**K**-SAnYG | 1319.3 | 25 | 448.8 -> 726.4 | 6.0 | 2.0 | 6.0 | 0.9907 |
| A1AG1 | NWGLSVYADKPETT**K**-SAnYG | 2140.2 | 600 | 573.0 -> 708.9 | 9.8 | 7.4 | 22.6 | 0.9902 |
| A1AG2 | SDVMYTDW**K**-SAnYG | 1575.6 | 200 | 576.8 -> 720.3 | 9.7 | 5.9 | 17.9 | 0.9965 |
|  | TLMFGSYLDDE**K**-SAnYG | 1849.9 | 200 | 713.8 -> 934.4 | 15.1 | 6.3 | 19.0 | 0.9993 |

# Table S-4. Metabolite internal standards for selected reaction monitoring assay, quantifier transitions, and validation parameters.

| **Analyte** | **Short name** | **Average MW** | **Spiked Metabolite Mixture (nM)** | **Quantified SRM Transition** | **Retention time (min)** | **calibration** | | |
| --- | --- | --- | --- | --- | --- | --- | --- | --- |
|  |  |  |  |  |  | **LOD (nmol/L)** | **LOQ (nmol/L)** | **R^2^** |
| Anthranilate | ATA | 137.1 | 200 | 138.1 -> 120.0 | 7.1 | 0.08 | 0.25 | 0.9998 |
| Indole-3-acetic acid | IAA | 175.2 | 200 | 176.1 -> 130.0 | 8.2 | 15 | 50 | 0.9986 |
| Indole-3-aldehyde | IAld | 145.2 | 200 | 146.1 -> 118.1 | 8.1 | 6 | 19 | - |
| Indole-3-acetamide | IAM | 174.2 | 50 | 175.1 -> 130.1 | 7.2 | 0.19 | 0.62 | 0.9999 |
| Indole-3-butyric-acid | IBA | 203.2 | 200 | 204.1 -> 186.0 | 9.0 | 4 | 14 | - |
| Indole-3-lactic acid | ILA | 205.2 | 200 | 206.1 -> 117.9 | 7.9 | 16 | 53 | - |
| Indole-3-propionic acid | IPA | 189.2 | 200 | 190.1 -> 130.1 | 8.7 | 6 | 20 | - |
| L-Kynurenine | KYN | 208.2 | 2000 | 209.1 -> 94.0 | 1.6 | 25 | 85 | 0.9996 |
| N-Acetyl-Tryptophan | NAT | 246.3 | 200 | 247.1 -> 188.1 | 8.2 | 4 | 13 | - |
| Tryptophan | TRP | 204.2 | 20000 | 205.1 -> 188.0 | 2.9 | 7.50 | 25.00 | 0.9979 |

Table S-5. Library for selected reaction monitoring metabolite assays**.** (*= quantifier transition)

| **Compound** | **Precursor** | **Product** | **RT [min]** |
| --- | --- | --- | --- |
| L-KYNURENINE* | 209.1 | 192.0 | 1.6 |
| L-KYNURENINE | 209.1 | 94.0 | 1.6 |
| [^2^D_4_] L-KYNURENINE* | 215.1 | 198.0 | 1.6 |
| [^2^D_4_] L-KYNURENINE | 215.1 | 96.1 | 1.6 |
| L-TRYPTOPHAN | 205.1 | 188.0 | 2.9 |
| L-TRYPTOPHAN* | 205.1 | 91.0 | 2.9 |
| [^13^C_11_] [^15^N_2_] L-TRYPTOPHAN | 218.1 | 200.0 | 2.9 |
| [^13^C_11_] [^15^N_2_] L-TRYPTOPHAN* | 218.1 | 98.1 | 2.9 |
| ANTHRANILIC ACID | 138.1 | 120.0 | 7.2 |
| ANTHRANILIC ACID* | 138.1 | 65.0 | 7.2 |
| [^13^C_6_] ANTHRANILIC ACID | 144.1 | 126.1 | 7.2 |
| [^13^C_6_] ANTHRANILIC ACID* | 144.1 | 70.2 | 7.2 |
| INDOLE-3-ACETAMIDE* | 175.1 | 130.1 | 7.3 |
| INDOLE-3-ACETAMIDE | 175.1 | 103.0 | 7.3 |
| [^2^H_5_] [^15^N] INDOLE-3-ACETAMIDE* | 181.1 | 134.1 | 7.3 |
| [^2^H_5_] [^15^N] INDOLE-3-ACETAMIDE | 181.1 | 106.0 | 7.3 |
| N-ACETYLTRYPTOPHAN* | 247.1 | 188.1 | 7.8 |
| N-ACETYLTRYPTOPHAN | 247.1 | 130.0 | 7.8 |
| N-ACETYLTRYPTOPHAN | 247.1 | 118.1 | 7.8 |
| INDOLE-3-LACTIC ACID | 206.1 | 188.0 | 7.8 |
| INDOLE-3-LACTIC ACID | 206.1 | 130.1 | 7.8 |
| INDOLE-3-LACTIC ACID* | 206.1 | 117.9 | 7.8 |
| INDOLE-3-ALDEHYDE* | 146.1 | 118.1 | 8.0 |
| INDOLE-3-ALDEHYDE | 146.1 | 117.1 | 8.0 |
| INDOLE-3-ALDEHYDE | 146.1 | 91.1 | 8.0 |
| INDOLE-3-ALDEHYDE | 146.1 | 65.1 | 8.0 |
| INDOLE-3-ALDEHYDE | 146.1 | 39.2 | 8.0 |
| INDOLE-3-ACETATE* | 176.1 | 130.1 | 8.2 |
| [^13^C_6_] INDOLE-3-ACETATE* | 182.1 | 136.1 | 8.2 |
| INDOLE-3-PROPIONIC ACID* | 190.1 | 130.1 | 8.6 |
| INDOLE-3-PROPIONIC ACID | 190.1 | 55.1 | 8.6 |
| INDOLE-3-BUTYRIC ACID* | 204.1 | 186.1 | 8.9 |
| INDOLE-3-BUTYRIC ACID | 204.1 | 168.0 | 8.9 |

# Table S-6. The concentration range for metabolite internal standards with linear response (linearity range).

| **Metabolite** | **Concentration Range [nM]** |
| --- | --- |
| IAA | 0.04 - 417640 |
| TRP | 0.08 - 750000 |
| KYN | 0.075 - 750000 |
| ATA | 0.000175 - 1750 |
| IAM | 0.0012 - 12000 |

# Figure S-1. Flowchart of dried blood specimen processing protocol.


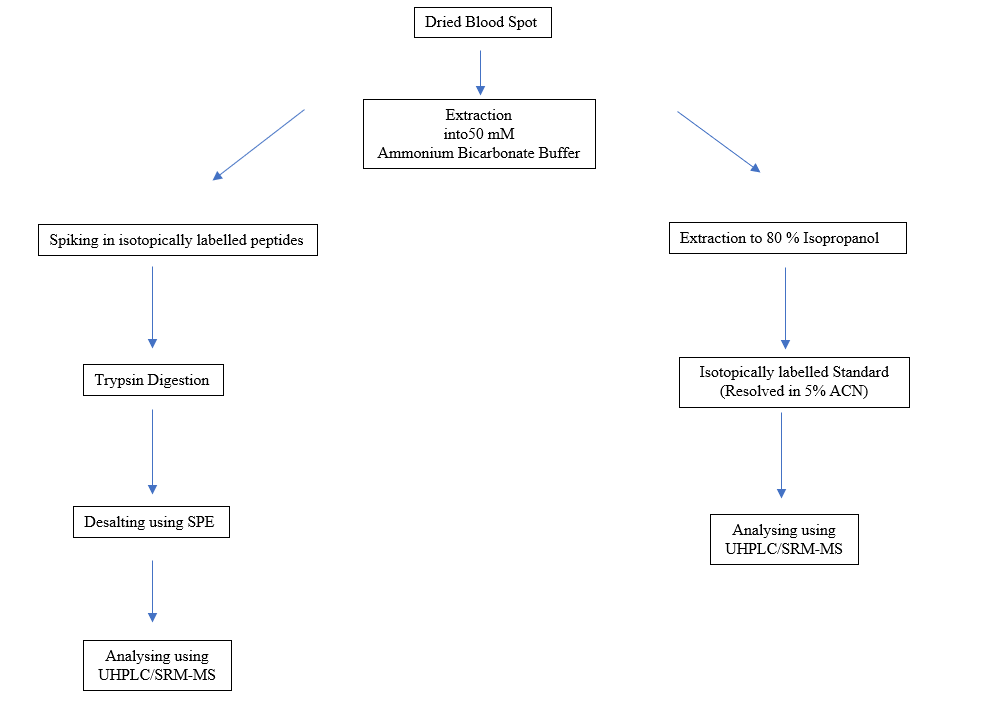


**Figure S-2.** Typical chromatograms of tryptophan catabolites in dried blood specimens. The upper panel shows the conformity with SRM signature and retention time of measured standards, the lower panel in measured DBS samples.


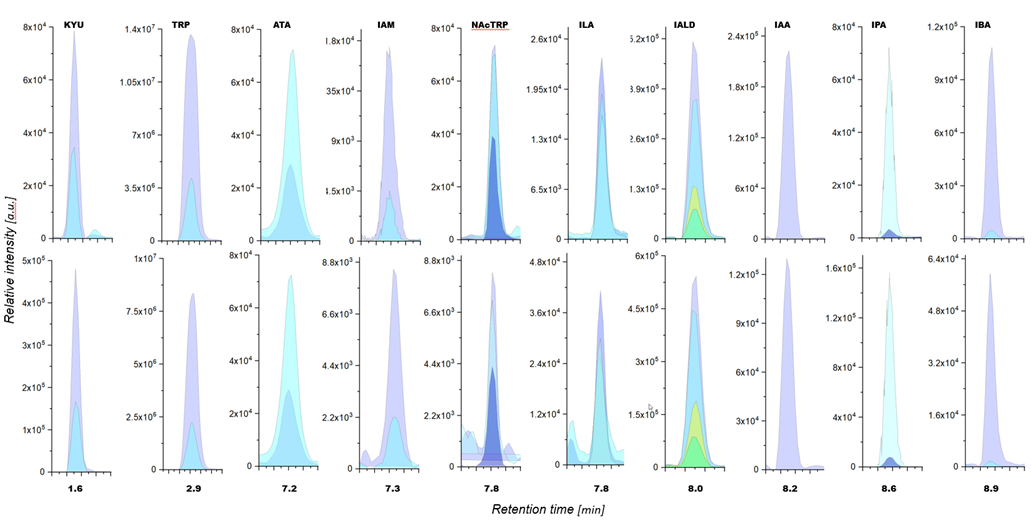


**Figure S-3.** Matrix-matched calibration curves for metabolite internal standards. Calibration curves in a matrix-matched solution with the addition of isotopically labeled anthranilate (ATA*), indole-3-acetic acid (IAA*), indole-3-acetamide (IAM*), kynurenine (KYN*), tryptophan (TRP*)

Figure S-4. Heatmap with metabolites and proteins quantified in dried blood specimens. The darker the red/blue color, the higher/lower is the measured concentration. The yellow/orange-colored inserted plot indicates the categorized analytes below/above LOD/LOQ (CRP, SAA1, IPA, IAM). The green/red colored inserted plot indicates the mode of delivery. The grey-colored inserted plot shows various anamnesis. Letters A-E indicate the resulting clusters.


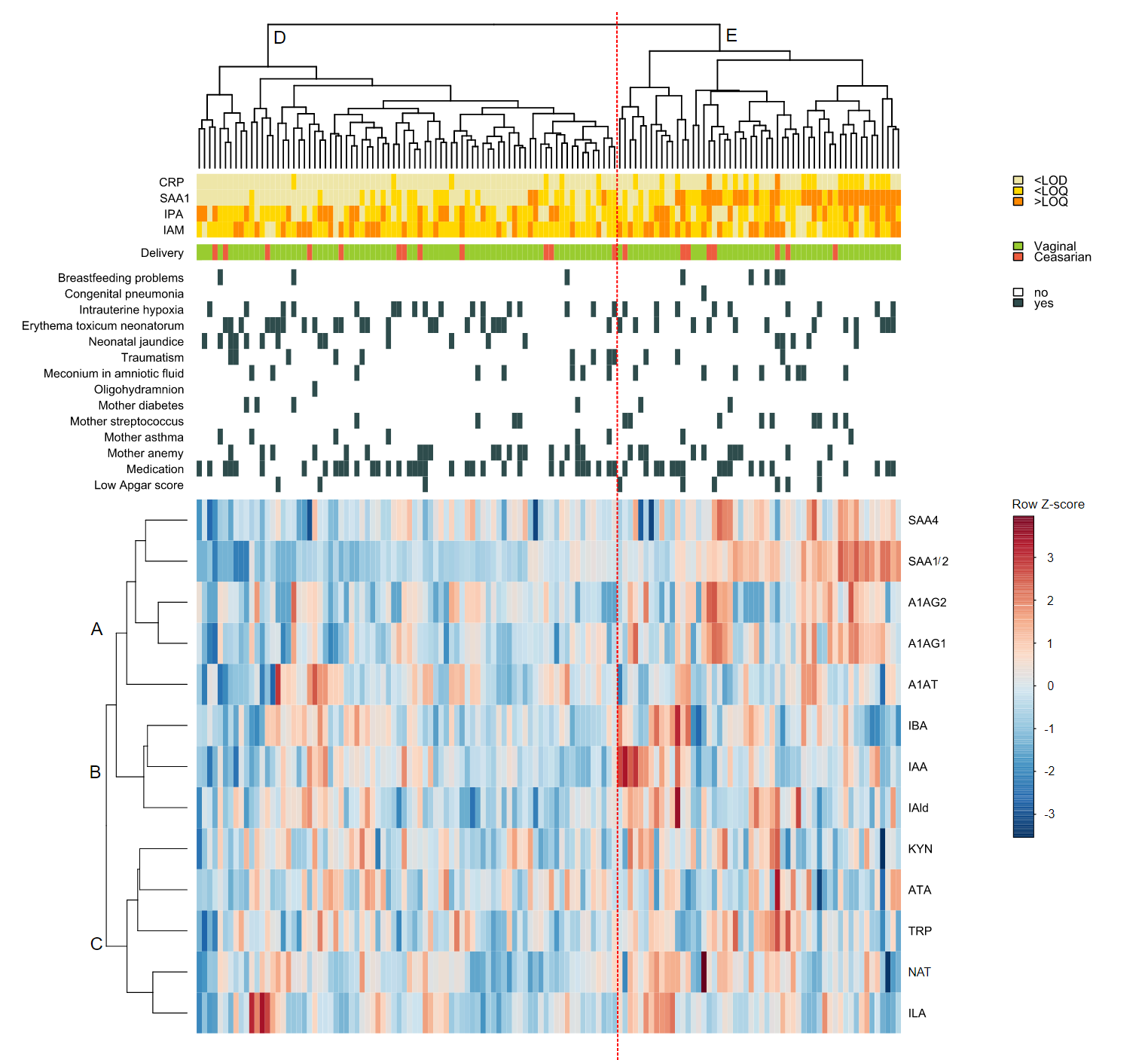


Figure S-5. Heatmap with proteins quantified in dried blood specimens. The darker the red/blue color, the higher/lower is the measured concentration. The yellow/orange-colored inserted plot indicates the categorized analytes below/above LOD/LOQ (CRP, SAA1, IPA, IAM). The green/red colored inserted plot indicates the mode of delivery. The grey-colored inserted plot shows various anamnesis. Letters A-E indicate the resulting clusters.


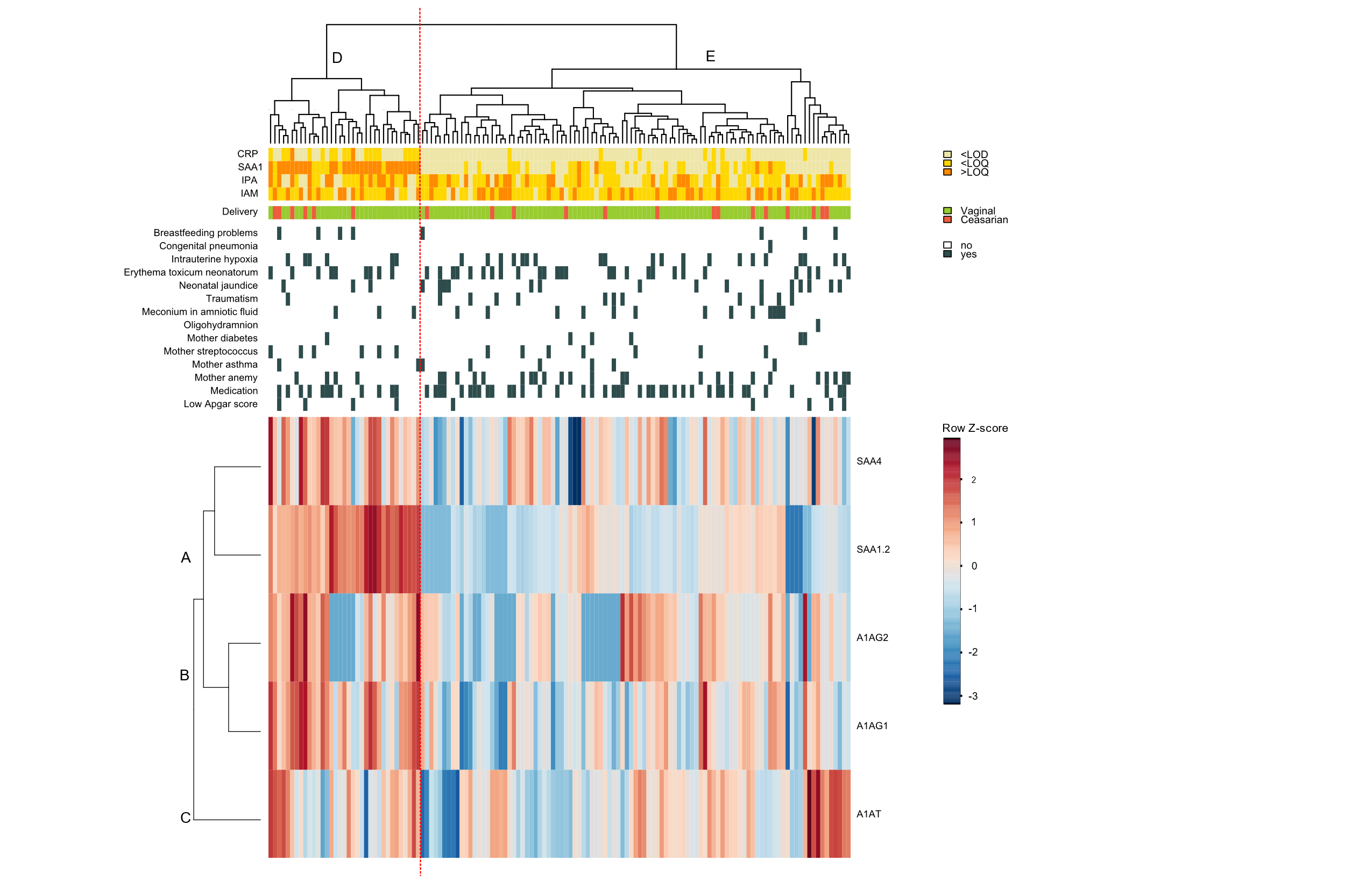


**Figure S-6. SAA1/2, SAA4, and A1AG1 blood concentrations are grouped relative to the limit of detection (LOD) for CRP (a) or SAA1 (b), p<0.0001.** Only SAA4 grouping in panel (b) was statistically significant (p<0.005). Sample concentrations >LOQ marked with black dots. Y-axis is in log-scale.


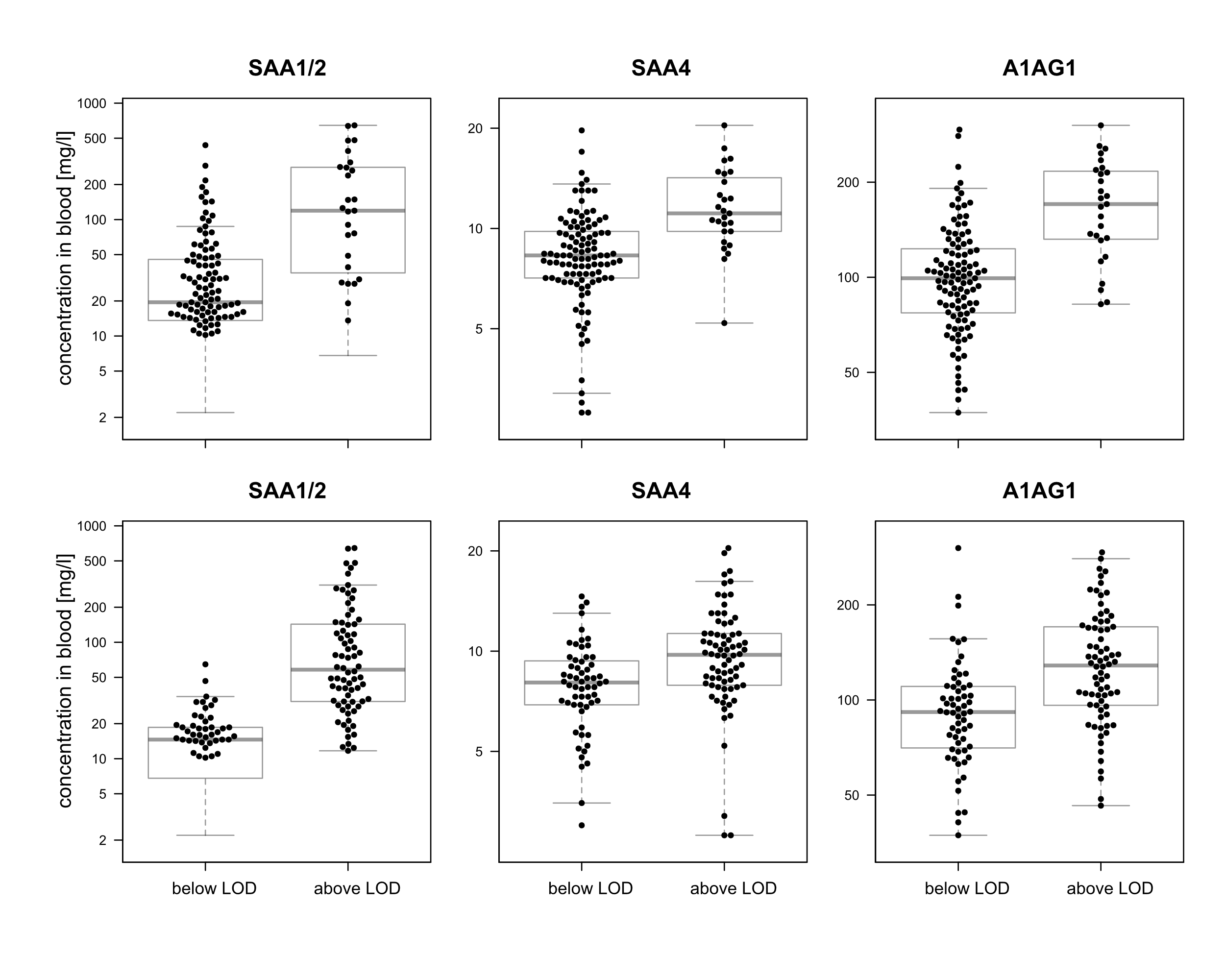


**a**

**b**

Figure S-7. Metabolites and proteins correlation matrix plots for vaginally (VD) and caesarian (CD) born neonates. Values of Pearson correlation coefficients are color-coded. A positive correlation is marked in blue and a negative correlation in red circles (the higher the Pearson correlation coefficient, the darker color, and the larger circles). The BH adjusted statistical significance is shown as stars. *** for *p* < 0.001, ** for *p* < 0.01 and * for *p* < 0.05.


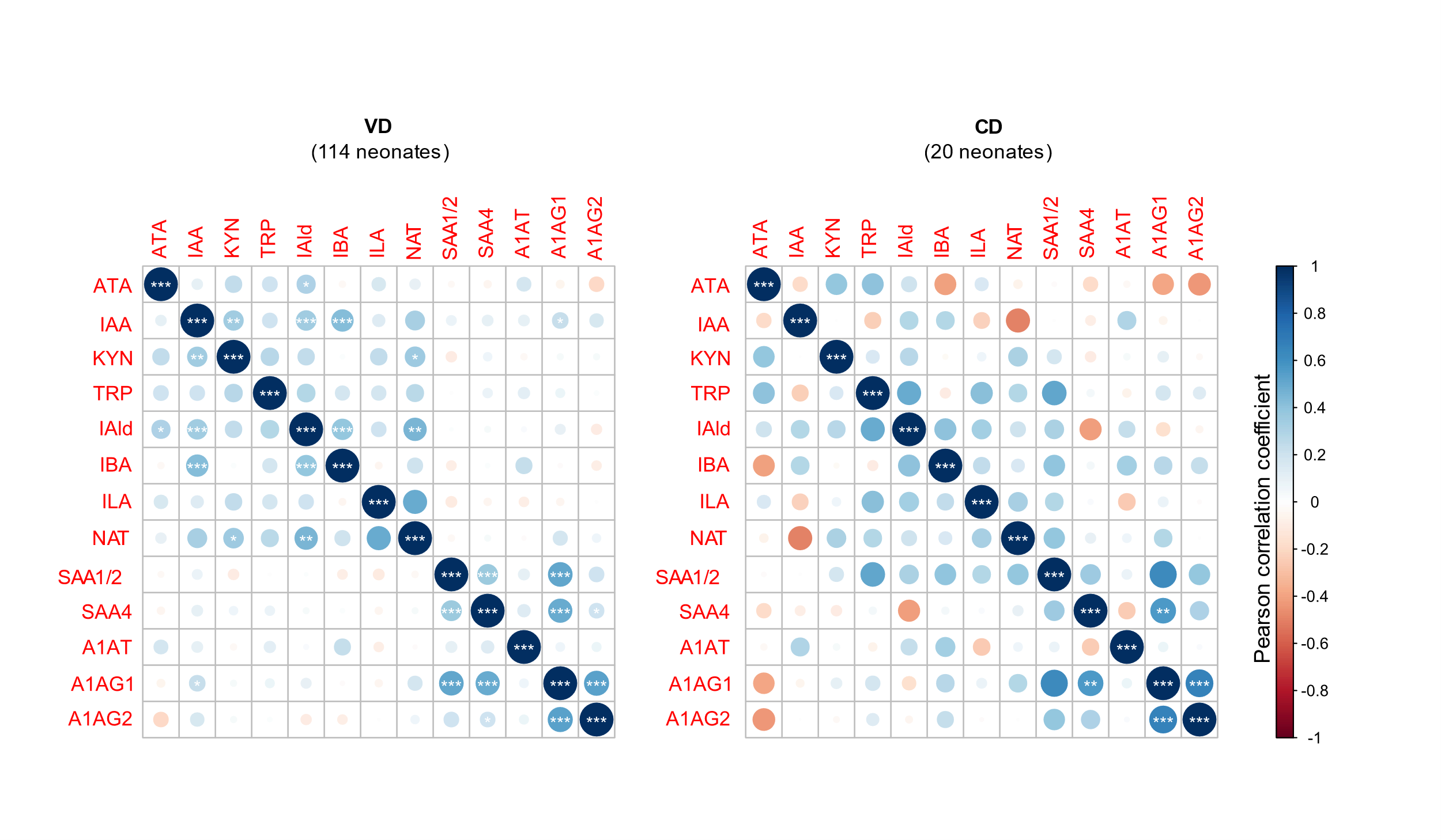


# Figure S-8. ILA and IAA correlation within all dried blood specimens and grouped by neonates' mode of delivery (*VD* for vaginal and *CD* for Cesarean delivery). R stands for the Pearson correlation coefficient.


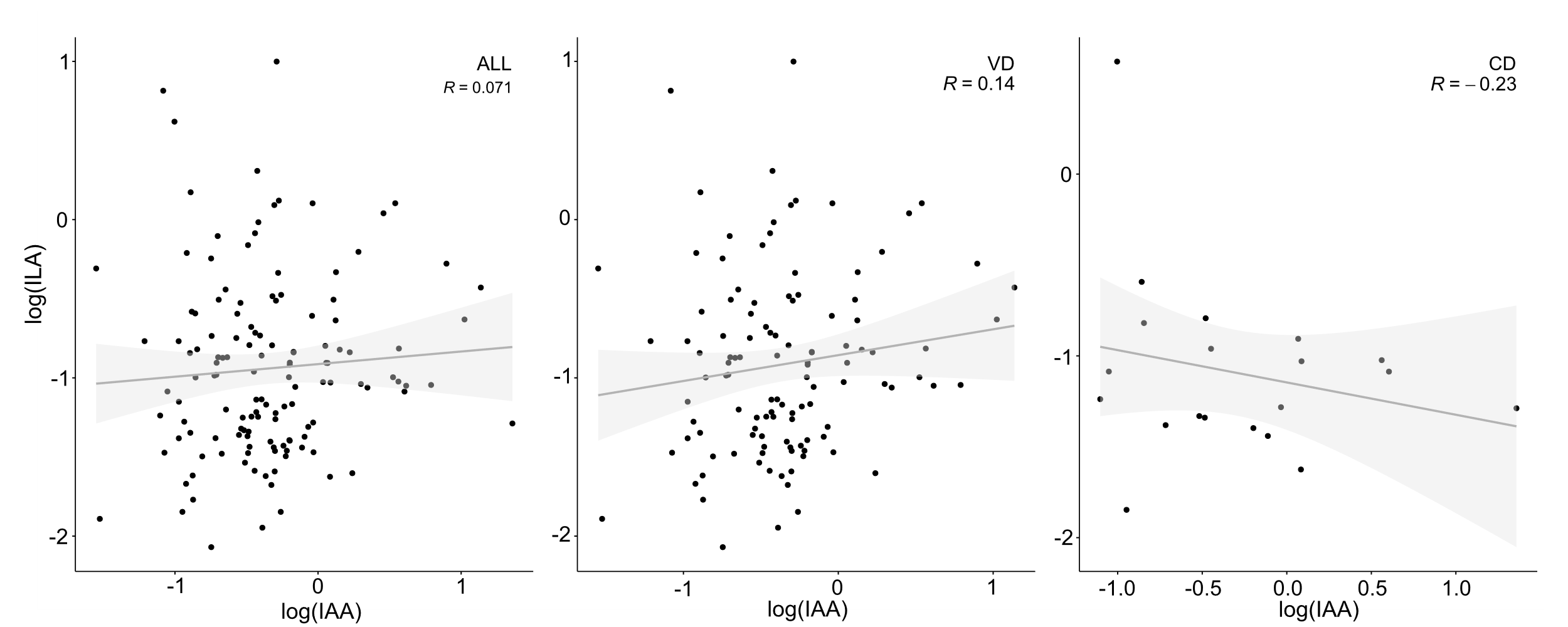

Supplement: Supplementary file 1 [file Data_Sheet_1.docx]
